# The Polar Organizing Protein PopZ Is Fundamental for Proper Cell Division and Segregation of Cellular Content in *Magnetospirillum gryphiswaldense*

**DOI:** 10.1128/mBio.02716-18

**Published:** 2019-03-12

**Authors:** Daniel Pfeiffer, Mauricio Toro-Nahuelpan, Marc Bramkamp, Jürgen M. Plitzko, Dirk Schüler

**Affiliations:** a Institute of Microbiology, University of Bayreuth, Bayreuth, Germany; b Department of Molecular Structural Biology, Max Planck Institute of Biochemistry, Planegg-Martinsried, Germany; c Department of Biology I, Ludwig-Maximilians-University Munich, Planegg-Martinsried, Germany; Nanyang Technological University

**Keywords:** magnetosome, *Magnetospirillum*, magnetotaxis, PopZ, polarity

## Abstract

Magnetotactic bacteria (MTB) are of special scientific interest due to the formation of magnetosomes, intracellular membrane-enveloped magnetite crystals arranged into a linear chain by a dedicated cytoskeleton. Magnetotaxis relies on the formation and proper inheritance of these unique magnetic organelles, both of which need to be coordinated with the segregation of other cellular content such as chromosomes or motility and chemotaxis related structures. Thus, elaborated mechanisms are required in MTB to coordinate and maintain a high level of spatial and temporal subcellular organization during cytokinesis. However, thus far, underlying mechanisms and polarity determinants such as landmark proteins remained obscure in MTB. Here, we analyzed an ortholog of the polar organizing protein Z in the alphaproteobacterium Magnetospirillum gryphiswaldense termed PopZ*_Mgr_*. We show that deletion of the *popZ_Mgr_* gene causes abnormal cell elongation, minicell formation, DNA missegregation, and impairs motility. Overproduction of PopZ*_Mgr_* results in PopZ-rich regions near the poles, which are devoid of larger macromolecules, such as ribosomes, chromosomal DNA, and polyhydroxybutyrate (PHB) granules. Using superresolution microscopy, we show that PopZ*_Mgr_* exhibits a bipolar localization pattern throughout the cell cycle, indicating that the definition of new poles in M. gryphiswaldense occurs immediately upon completion of cytokinesis. Moreover, substitution of PopZ orthologs between M. gryphiswaldense and the related alphaproteobacterium Caulobacter crescentus indicated that PopZ localization depends on host-specific cues and that both orthologs have diverged to an extent that allows only partial reciprocal functional complementation. Altogether, our results indicate that in M. gryphiswaldense, PopZ plays a critical role during cell division and segregation of cellular content.

## Abstract (precis)

Magnetotactic bacteria (MTB) share the unique capability of magnetic navigation, one of the most complex behavioral responses found in prokaryotes, by means of magnetosomes, which act as an internal compass. Due to formation of these unique nanoparticles, MTB have emerged as a model to study prokaryotic organelle formation and cytoskeletal organization in conjunction with complex motility systems. Despite the high degree of subcellular organization required in MTB, less is known about cell-cycle-related factors or proteins responsible for spatiotemporal polarity control. Here, we investigate the function of the polar organizer PopZ in the magnetotactic alphaproteobacterium Magnetospirillum gryphiswaldense. Although PopZ is widely distributed among the alphaproteobacteria, its function in MTB belonging to this class has remained unexplored. Our results suggest that in M. gryphiswaldense, PopZ has a key role during cell division and subcellular organization. Furthermore, we show that PopZ localization and function differ from other nonmagnetotactic alphaproteobacterial model organisms.

## INTRODUCTION

During cytokinesis, bacteria have to coordinate division with the equipartitioning or *de novo* synthesis of cellular content, such as chromosomes, intracellular storage granules, or (polar) structures, such as chemosensory clusters, flagella, or pili (1–5). In magnetotactic bacteria (MTB), this in addition has to be coordinated with the proper duplication and segregation of their key organelles, the magnetosomes (6–8). In the widely studied magnetotactic alphaproteobacterium Magnetospirillum gryphiswaldense, the magnetosomes consist of membrane-enclosed crystals of magnetite, which during their biosynthesis become arranged into linear chains to build a magnetic sensor sufficiently strong to align the cells in the weak Earth’s magnetic field (9, 10). During cell division, this magnetosome chain is then split and magnetosomes are equipartitioned to daughter cells (6, 7). To overcome magnetic forces during separation of magnetosome chains, cells divide by asymmetric septation (i.e., unidirectional constriction of the inner and outer membranes) (6). Both magnetosome chain formation and division are orchestrated by a multipartite and complex cytoskeleton (the “magnetoskeleton” [M. Toro-Nahuelpan, G. Giacomelli, O. Raschdorf, S. Borg, J. M. Plitzko, M. Bramkamp, D. Schüler, and F. D. Müller, submitted for publication]), involving the actin-like MamK protein, which forms dynamic filaments which position and relocate the magnetosome chain within the cell (11, 12). For proper magnetic navigation, the inherent magnetic polarity of the resulting cellular compass has to be coordinated with the organization of motility and chemotaxis structures, such as polar flagella and chemosensory clusters, resulting in a biased directionality of swimming motility (8, 13). However, despite the high degree of intra- and extracellular polar organization required in MTB, underlying cell-cycle-related factors and mechanisms have been only poorly characterized.

In other alphaproteobacteria, polar organizing protein Z (PopZ) has been found to play a major role in definition of cell polarity and spatiotemporal control of multiple proteins. In Caulobacter crescentus, PopZ (PopZ*_Cc_*) assembles into a putative filamentous network in chromosome-free regions at the cell poles, serving as a hub for many other cell-cycle-related proteins (14–18). During the asymmetric cell cycle of C. crescentus, PopZ first localizes to the old stalked pole, where it binds to the adaptor protein ParB, tethering the chromosome to the pole. In the second half of the cell cycle, the PopZ network duplicates at the opposite pole, to ensure capture of the sister chromosome upon replication, which avoids chromosome missegregation (17, 18). In addition, PopZ plays a critical role in cell division by polar retention of MipZ, which is a regulator ensuring proper midcell assembly of the FtsZ ring (19, 20). Therefore, deletion of *popZ* in C. crescentus causes severe cell division phenotypes, including abnormal cell elongation and formation of minicells (17, 18). Additionally, since PopZ is required for robust recruitment of proteins necessary for stalk synthesis, loss of *popZ* also affects formation of this organelle (15). In Agrobacterium tumefaciens, which grows predominantly by addition of peptidoglycan at one pole (the new “growth pole”) (21, 22), PopZ is located specifically at the growing pole. After septation, PopZ switches to the newly generated “growth poles” of both daughter cells (23, 24). Absence of *popZ* in A. tumefaciens resulted in asymmetric sites of cell constriction and cell branching (25, 26). In the pathogen Brucella abortus, PopZ is also unipolar and marks the new pole throughout the cell cycle (27).

Although PopZ is widespread among the alphaproteobacteria (28), its function has been studied in only a very few representatives, and its role in MTB from this class has remained entirely unexplored. Here, we characterized an ortholog of the PopZ polarity factor in M. gryphiswaldense (referred to as PopZ*_Mgr_*). Loss of *popZ_Mgr_* caused severe defects of growth, cell division, and motility. In contrast, the dynamic MamK pole-to-midcell treadmilling was independent of PopZ*_Mgr_*. Moreover, we observed remarkable differences in the cell-cycle-dependent localization pattern of PopZ between M. gryphiswaldense, C. crescentus, and A. tumefaciens. Substitution of PopZ orthologs between M. gryphiswaldense and C. crescentus indicated that the proteins from both strains can partially replace their respective functionalities, but differ to an extent that prevents full implementation within the cell-cycle-dependent interaction network of the heterologous host. In summary, our results reveal a key role of PopZ*_Mgr_* in subcellular organization and provide the first fundamental insights into its function in cell cycle control and polarity determination in MTB. Thus, this work also demonstrates the usefulness of M. gryphiswaldense as a potential and emerging model to scrutinize the bacterial cell cycle and its coordination to spatiotemporal organelle organization.

## RESULTS

### PopZ*_Mgr_* localizes to both cell poles.

**FIG 1:**
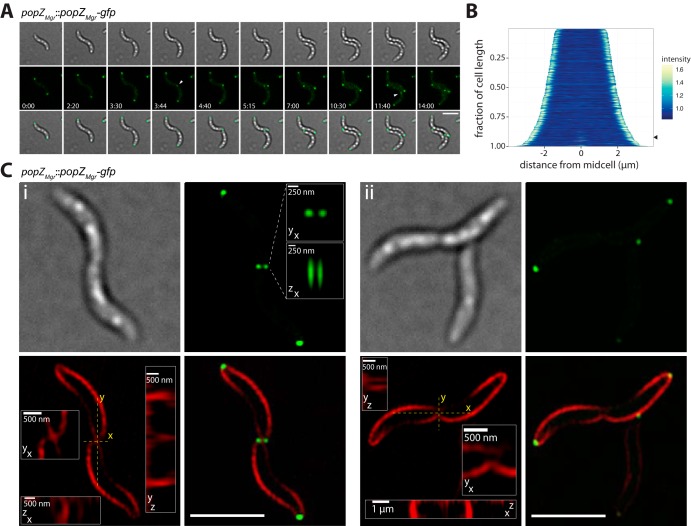
Localization of PopZ*_Mgr_*-GFP in M. gryphiswaldense. (A) Time-lapse microscopy of cells expressing PopZ*_Mgr_*-GFP (*popZ_Mgr_*::*popZ_Mgr_-gfp* strain). First row, bright-field; second row, fluorescence channel; third row, overlay of bright-field and GFP channel. PopZ_*Mgr*_ localizes to both cell poles. In dividing cells, an additional spot appears at the cell division site (fourth and ninth frames, white arrowheads). Generation time during time-lapse series was approximately 4 to 5 h. Numbers indicate hours and minutes. (B) Demograph of cells expressing PopZ*_Mgr_*-GFP (*n* = 642 cells). The appearance of the signal at midcell is marked with an arrowhead. (C) Structured illumination microscopy (3D-SIM) allows us to resolve two PopZ foci in close proximity at the division plane with a distance near the resolution limit of conventional epifluorescence microscopy (∼250 nm). Micrographs are maximum-intensity projections of z-stack images from representative FM4-64-stained dividing cells. First row, bright-field (left image) and GFP channel (right image); second row, FM4-64 channel (left image) and overlay of FM4-64 and GFP channel (right image). Insets are magnified xy, xz, and yz projections of PopZ foci (GFP channel) and division plane (FM4-64 channel). Note cells with PopZ foci present at the site of division (Ci) had already completed compartmentalization and separation of their membranes, whereas no foci were observed in cells that were still connected (Cii). A representative number of dividing cells was imaged (*n* = 29 [additional cells are shown in Fig. S2]). All scale bars not indicated in the figure correspond to 3 μm.

In M. gryphiswaldense, PopZ*_Mgr_* is encoded in a conserved genomic region, similar to C. crescentus, next to putative genes coding for a valyl-tRNA synthetase and an outer membrane efflux protein (see Fig. S1A in the supplemental material). To study its localization pattern throughout the cell cycle, PopZ*_Mgr_* was translationally fused to green fluorescent protein (GFP) by integration of an M. gryphiswaldense codon-optimized *gfp* gene (*magegfp* [29]) within this genomic region. Expression of *popZ_Mgr_-gfp* from its native promoter was verified via immunoblotting using an antibody against GFP (not shown). *In vivo* time-lapse fluorescence microscopy revealed that PopZ*_Mgr_*-GFP localized to both cell poles and exhibited a bipolar localization pattern throughout the cell cycle (Fig. 1A; see Movie S1 in the supplemental material). PopZ foci at the future new poles appeared at the end of the cell cycle (Fig. 1B). Since conventional wide-field microscopy did not allow us to judge with high confidence if cells with PopZ foci present at the division plane had already completed cytokinesis, we imaged dividing cells with superresolution three-dimensional structured illumination microscopy (3D-SIM). Using FM4-64 membrane staining, 3D-SIM revealed two adjacent PopZ foci (∼250 nm apart) at the cell division site (Fig. 1Ci; see Fig. S2 in the supplemental material). In general, all cells with two PopZ foci present at the division site had already completed separation of their membranes (Fig. 1Ci and Fig. S2). In contrast, no PopZ foci were observed in cells with membranes and cytoplasm still connected, but which already had undergone partial membrane constriction (Fig. 1Cii and Fig. S2). These results indicated that formation of PopZ-rich zones at the new poles occurs very late during or shortly after completion of cytokinesis.

10.1128/mBio.02716-18.1 FIG S1 Genomic locus, secondary structure of PopZ*_Mgr_*, and global alignment of different PopZ orthologs. (A) The *popZ_Mgr_* gene (red) is located next to putative genes coding for an outer membrane efflux protein and a valyl-tRNA synthetase. (B) PopZ*_Mgr_* secondary structure prediction with EMBOSS Protein Analysis Tools, version 1.0. Alpha-helices (green), beta-strands (yellow), turns (blue), and DUF2497 (= domain of unknown function) are indicated. PopZ*_Mgr_* has a theoretical length of 226 amino acids and molecular mass of 25.35 kDa. The overall protein was predicted to be cytoplasmatic (TMHMM Transmembrane Prediction Tool, version: 0.9). The central region of PopZ*_Mgr_* is characterized by a high content of proline and glutamic acid residues (15.5% and 16.4% of total amino acids, respectively) arranged in repetitive patterns. (C) Multiple sequence alignment generated with Geneious 8.1.9 (BLOSUM62 similarity matrix) of different PopZ orthologs in Magnetospirillum gryphiswaldense MSR-1 (Mgr_3089), Caulobacter crescentus CB15 (CC_1319), Magnetospirillum magneticum AMB-1 (AMB_2246), Magnetospirillum magnetotacticum MS-1 (CCC_02167), Phaeospirillum fulvum MGU-K5 (WP_021131188), Phaeospirillum molischianum DSM120 (WP_002726807), Rhodospirillum rubrum ATCC 11170 (RRU_A1797), Magnetospira sp. strain QH-2 (MGMAQ_1523), and Azospirillum brasilense Az39 (ABAZ39_06655). The Mgr_3089, RRU_A1797, and WP_002726807 sequences were corrected by the first 11 amino acids (42 amino acids for WP_002726807) missing in the originally annotated sequences. Amino acids are colored according to their similarity. PopZ orthologs are well conserved in their N-terminal and C-terminal regions, both of which are predicted to form α-helices by secondary structure analysis. The C-terminal region has been previously shown to be necessary for polar localization in C. crescentus, presumably by promoting the oligomerization of PopZ by direct interaction between PopZ molecules, whereas the N-terminal region is important for an interaction with the chromosome segregation ATPase ParA and adaptor protein ParB (G. Laloux and C. Jacobs-Wagner, J Cell Biol 201:827–841, 2013, https://doi.org/10.1083/jcb.201303036; G. R. Bowman, A. M. Perez, J. L. Ptacin, E. Ighodaro, E. Folta-Stogniew, L. R. Comolli, and L. Shapiro, Mol Microbiol 90:776–795, 2013, https://doi.org/10.1111/mmi.12398). In contrast, recent studies of PopZ in C. crescentus suggest that the central proline-rich region, which is less conserved in sequence and length among different PopZ orthologs and enlarged in PopZ from different magnetotactic bacteria, behaves more like a linker than harboring its own distinct function (J. A. Holmes, S. E. Follett, H. Wang, C. P. Meadows, K. Varga, and G. R. Bowman, Proc Natl Acad Sci U S A 113:12490–12495, 2016, https://doi.org/10.1073/pnas.1602380113). (D) Pairwise sequence identity (above the diagonal of 100 % values) and similarity (below the diagonal) calculated with SIAS (http://imed.med.ucm.es/Tools/sias.html) from the multiple-sequence alignment shown in panel C. The identity was calculated as the number of identical positions divided by the mean length of sequences. Download FIG S1, PDF file, 2.6 MB. Copyright © 2019 Pfeiffer et al. 2019 Pfeiffer et al. This content is distributed under the terms of the Creative Commons Attribution 4.0 International license.

10.1128/mBio.02716-18.2 FIG S2 Structured illumination microscopy (3D-SIM) of FM4-64-stained dividing cells expressing PopZ*_Mgr_*-GFP (*popZ_Mgr_*::*popZ_Mgr_*-*gfp* strain). From left to right are shown the bright-field, FM4-64 channel, GFP channel, and FM4-64 plus GFP overlay. Fluorescence micrographs are maximum-intensity projections of z-stacks. Putative outer membrane vesicles (OMV) and spheroblasts are marked with white arrowheads. (Third column, last row) Cell dividing during imaging. The FM4-64 channel was imaged first. Note two PopZ foci visible at the cell division site were only observed in cells that had completed separation of their membranes. Scale bars = 2 µm. Download FIG S2, PDF file, 2.4 MB. Copyright © 2019 Pfeiffer et al. 2019 Pfeiffer et al. This content is distributed under the terms of the Creative Commons Attribution 4.0 International license.

10.1128/mBio.02716-18.7 MOVIE S1 Time-lapse microscopy of the M. gryphiswaldense *popZ_Mgr_*::*popZ_Mgr_-gfp*, wild-type, and Δ*popZ_Mgr_* strains. Time and strain are indicated in the upper left and upper right corners, respectively. One second of playback time corresponds to 105 min (*popZ_Mgr_*::*popZ_Mgr_-gfp* strain) or 60 min (wild-type and Δ*popZ_Mgr_* strains). Download Movie S1, AVI file, 10.0 MB. Copyright © 2019 Pfeiffer et al. 2019 Pfeiffer et al. This content is distributed under the terms of the Creative Commons Attribution 4.0 International license.

### Deletion of *popZ_Mgr_* causes severe cell division defects.

**FIG 2:**
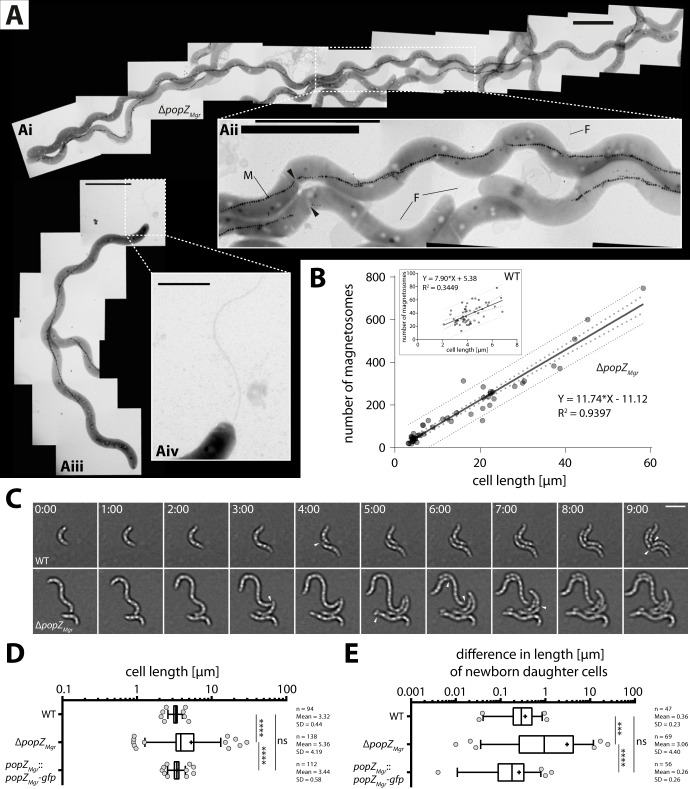
Deletion of *popZ* in M. gryphiswaldense causes severe cell division defects. (A) Upon deletion of *popZ_Mgr_*, highly elongated cells (up to 60 µm) were observed, which contained long, sometimes interrupted (black arrowheads) magnetosome chains (M) traversing throughout the cell, except polar regions (Ai to Aiii). Bipolar positioning of flagella (F) was not affected in the Δ*popZ_Mgr_* strain (Aiii and Aiv), but occasionally additional flagella were observed at ectopic positions along the cell’s body (Ai and Aii). Scale bars correspond to 1 μm (Aii and Aiv) and 3 µm (Ai and Aiii). (B) A linear correlation between cell length and number of magnetosomes was revealed for the Δ*popZ_Mgr_* strain, including highly elongated cells, with single chains consisting of more than 700 particles. Dotted lines indicate 95% confidence intervals (big dots) and prediction intervals (small dots). A total number of 47 cells for the Δ*popZ_Mgr_* strain and 54 cells for the wild type (WT [inset]) were analyzed from at least 3 independent cultures grown under standard microoxic conditions. (C) Time-lapse microscopy of the M. gryphiswaldense wild-type and Δ*popZ_Mgr_* strains. Division of the Δ*popZ_Mgr_* cell occurs at ectopic positions. Cell division events are marked with white arrowheads. Time values are given in hours and minutes. Scale bar = 3 µm. (D) Cell length distributions and (E) difference in length of both newborn daughter cells of M. gryphiswaldense wild-type, Δ*popZ_Mgr_*, and *popZ_Mgr_*::*popZ_Mgr_-gfp* strains measured from time-lapse series. Note that the cell length distribution of the *popZ_Mgr_*::*popZ_Mgr_-gfp* strain, as well as growth (not shown), did not differ noticeably from those of the wild type (specific growth rates determined under oxic conditions at 28°C: *popZ_Mgr_*::*popZ_Mgr_-gfp* strain, 0.169 ± 0.002 h^−1^; wild type, 0.171 ± 0.006 h^−1^). In contrast, for the Δ*popZ_Mgr_* strain, which exhibited cells with high variation in length, a prolonged lag phase and decreased specific growth rate of 0.140 ± 0.021 h^−1^ were seen. In box plots, the bar indicates the median, the box the interquartile range, and the whiskers the 5th and 95th percentiles. The mean is shown as +. The number of measured cells (*n*) and mean and standard deviation (SD) are indicated in the graph. *P* values were determined by Kruskal-Wallis test with Dunn’s multiple-comparison posttest. *, *P* < 0.05, **, *P* < 0.01, ***, *P* < 0.001, ****, *P* < 0.0001; ns, not significant (*P* ≥ 0.05).

To study the effects of *popZ* absence in M. gryphiswaldense, a markerless in-frame deletion mutant was constructed. The Δ*popZ_Mgr_* strain was viable, but showed severely impaired growth and increased cell length (Fig. 2). Some cells were elongated up to 60 µm (Fig. 2Ai), equivalent to ∼20-fold the length of a newborn wild-type cell (∼3 µm). Elongated cells contained between 1 and 3 abnormally long magnetosome chains running in parallel, which were sometimes interspaced by segments of unknown origin and composition—i.e., parts of the cell body that appeared brighter in the electron microscope (Fig. 2Aii, black arrowheads). Elongated magnetosome chains were up to ∼20 µm in length (allowing gaps not larger than 150 nm). Elongated cells with more than 700 particles were observed; however, cell length and number of magnetosomes were well correlated, resulting in ∼12 particles/µm (Fig. 2B). For comparison, we determined that the wild type typically exhibited a median chain length of ∼1 µm and median magnetosome numbers of 35 particles per cell. In contrast to the Δ*popZ_Mgr_* strain, particle number and cell length were only poorly correlated in the wild type (Fig. 2B, inset), similar to previous correlative estimations of magnetosome particle numbers versus cell area (30). Moreover, even in the most highly elongated cells, magnetosome chains were mostly absent from the regions near the cell poles (Fig. 2Aiii). As is also commonly observed in the wild type (31), cells of the Δ*popZ_Mgr_* strain contained large amounts of polyphosphate and polyhydroxybutyrate (PHB) granules (Fig. 2Ai to Aiii).

During time-lapse microscopy (Fig. 2C), the wild type divided at a median cell length of 3.3 µm, whereas in the Δ*popZ_Mgr_* strain, the cell elongation and unequal division produced a much broader length distribution with a median cell length of 3.9 µm during division (Fig. 2D). Furthermore, the time required to complete a division cycle was less regular for the Δ*popZ_Mgr_* strain (Fig. 2C and Movie S1). While wild-type cells divide approximately every 4 to 5 h, cell division in the Δ*popZ_Mgr_* strain occurred with variable timing and at ectopic positions, suggesting that the generation time as well as division septum positioning were affected. To measure potential asymmetry during cell division, the difference in length of both newborn daughter cells was calculated from time-lapse series (Fig. 2E). Thus, the median cell length differences for wild-type daughter cells were 0.29 and 0.94 µm for the *popZ_Mgr_* deletion strain, confirming that the Δ*popZ_Mgr_* population contained cells with high variation in length caused by unequal division. Also, a slight asymmetry (∼11% off-center) was detected for newborn daughter cells of the wild-type strain, in agreement with previous observations (on average, 15% off-center) by Katzmann et al. (6).

### CET ultrastructural analysis of the Δ*popZ_Mgr_* mutant reveals missegregation of cellular content and chemotactic receptor arrays, septum mislocalization, and minicell formation.

**FIG 3:**
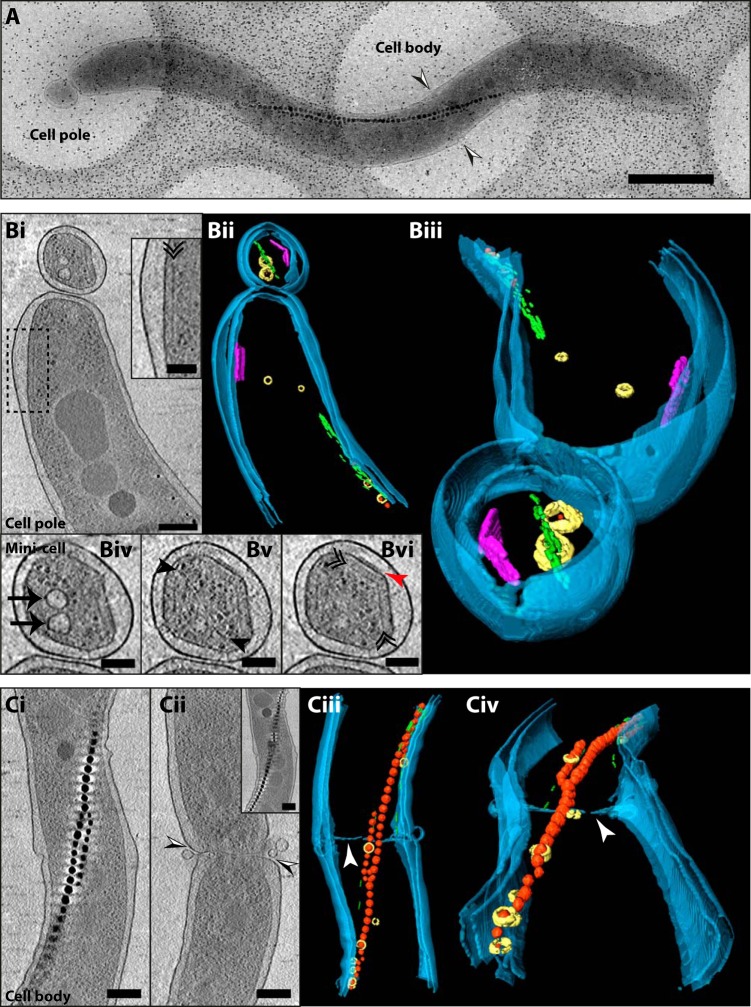
Cryo-electron tomography of the Δ*popZ_Mgr_* strain. (A) Cryo-electron micrograph of an elongated cell of the Δ*popZ_Mgr_* strain. Tomograms were acquired at the cell pole and cell body areas. Combined black and white arrowheads indicate the position of membrane invagination observed in panel Cii. Scale bar = 1 µm. (B) CET of the cell pole area. (Bi) A 15.7-nm-thick tomographic slice (average of 5 slices) through the central part of the cell. The black dashed rectangle indicates the area seen in the inset. (Inset) Base plate layer of a chemoreceptor array indicated by a black double arrowhead. (Bii and Biii) Three-dimensional rendering of the cell pole shown in panels A and Bi. Magnetite crystals are red, magnetosome membrane vesicles are yellow, the actin-like MamK filament is green, and the chemoreceptor arrays are purple. The cellular envelope inner and outer membranes are depicted in blue. (Biv) A 15.7-nm-thick tomographic slice of the minicell displaying magnetosome vesicles (black arrows), (Bv) the MamK filament (3.1-nm-thick slice [black arrowheads]), and (Bvi) the chemoreceptor array (red arrowhead, periplasmic chemoreceptor domains; black double arrowhead, chemoreceptor base plate layer). (C) CET of the cell body area: (Ci) A single 3.1-nm-thick tomographic slice through the central part of the cell displaying the magnetosome chain (electron-dense magnetite crystals arranged into a chain). (Cii) A 3.1-nm-thick slice near the cell edge showing a deep membrane unidirectional constriction at the cell body area. (Inset) A 3.1-nm slice in a different z position through the tomogram showing the continuity of the magnetosome chain from panel Ci. (Ciii and Civ) Three-dimensional rendering of the cell body area shown in panels A and Ci. White arrowheads show membrane invagination seen in panel Cii. Scale bars: panels Bi, Ci, and Cii = 200 nm; panels Biv to Bvi and insets = 100 nm. A total of 6 cells were analyzed.

Prompted by the observed cell elongation and impairment of division, we performed cryo-electron tomography (CET) to further investigate the cell division defect of the Δ*popZ_Mgr_* strain. A cryo-electron micrograph of an elongated Δ*popZ_Mgr_* cell (Fig. 3A) indicates the areas analyzed by CET (cell pole and cell body) and confirms the formation of distinct minicells at the poles that were also observed in time-lapse imaging experiments (Fig. 2C and Movie S1). CET and further segmentation of the cell pole showed the typical arrangement of magnetosomes by the actin-like MamK filament (Fig. 3Bi and Bii). In addition, a chemoreceptor array was observed at the pole close to the lateral cytoplasmic membrane (inset from Fig. 3Bi and purple objects in Bii), as observed previously in wild-type cells (11). Additionally, a tomographic slice of the cell pole revealed the presence of chemoreceptor arrays also within the minicell (Fig. 3Bi and vi), highlighted in the tomogram segmentation (purple objects in Fig. 3Bii and Biii). Moreover, additional structures inside the minicell, such as magnetosome membrane vesicles, MamK filaments, and ribosomes, were visible (Fig. 3Biv to vi), indicating putative defects in segregation of cellular content. The presence of chemoreceptor arrays at both the pole and within the adjacent minicell (Fig. 3B), and the observation of double chemoreceptor arrays at the cell poles (see Fig. S3Ai in the supplemental material) argue for an improper polar chemoreceptor array localization (CET micrographs of chemoreceptor arrays in the wild-type strain can be found in reference 11 for comparison).

10.1128/mBio.02716-18.3 FIG S3 Cryo-electron tomography of Δ*popZ_Mgr_* cells. Tomograms of all additional Δ*popZ_Mgr_* cells are shown (total *n* = 6). (A) Tomographic slices (15.7 nm thick) through the tomogram of (Ai and Aii) the cell pole and (Aiii and Aiv) cell body of an elongated Δ*popZ_Mgr_* cell (cell 2). (Aii and Aiv) Membrane constrictions are observed at the cell pole and cell body and therefore located far off midcell. Black and white arrowheads indicate membrane invagination. PP, polyphosphate granule; PHB, polyhydroxybutyrate granule; red arrowhead, periplasmic chemoreceptor domains; black double arrowheads, chemoreceptor base plate layer; black arrows, magnetosome vesicles. (B) Tomographic slices (15.7 nm thick) through the tomogram of a cell pole (cell 3) and a cell body (cell 4) of two different cells. (Bi and Bii) Cell 4 displays two deep membrane invaginations or unidirectional constrictions at different locations far off midcell (combined black and white arrowheads). Black arrowheads, MamK filaments; black arrows, magnetosome vesicles. (Biii) A 15.7-nm thick tomographic slice through the central part of a minicell from cell 3. (Ci) A 15.7-nm-thick tomographic slice through the center of the tomogram of a cell pole (cell 5). The black dashed rectangle indicates the area seen in the inset. (Inset) Base plate layer of a chemoreceptor array denoted by a black double arrowhead and the periplasmic chemoreceptor domains indicated by a red arrowhead. (Cii) Membrane constrictions observed at the cell pole located far off midcell (black and white arrowheads). (D) A 15.7-nm-thick tomographic slice through the center of the tomogram of the cell pole of cell 6. The black double arrowheads denote the chemoreceptor base plate layer. Scale bars = 200 nm in panel Biii, and the inset in panel Ci = 100 nm. Download FIG S3, JPG file, 2.7 MB. Copyright © 2019 Pfeiffer et al. 2019 Pfeiffer et al. This content is distributed under the terms of the Creative Commons Attribution 4.0 International license.

Localization of magnetosome chains did not seem to be affected in the Δ*popZ_Mgr_* strain, as tomographic slices of the cell body showed a properly localized magnetosome chain across the long cell axis (Fig. 3Ci). Remarkably, a deep unidirectional constriction of the membrane located distant from midcell indicated a putative septum mispositioning (black-white arrowheads in Fig. 3A and Cii and white arrowheads in Fig. 3Ciii and Civ). Furthermore, both the misplaced septum invaginations far-off midcell (Fig. S3Aii and Aiv and Bi, Bii, and Cii) and minicell formation (Fig. S3Bi to Biii) were commonly observed by CET. Therefore, minicell formation is likely caused by ectopic septum localization, confirming the cell division impairment in the Δ*popZ_Mgr_* mutant. Thus, Δ*popZ_Mgr_* cells are likely unable to properly control the FtsZ ring localization.

**FIG 4:**
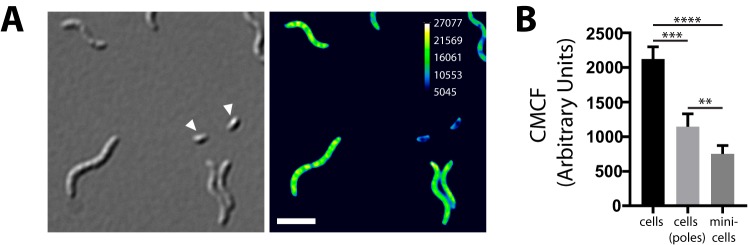
Minicells contain less or no DNA. (A) Micrographs of DAPI-stained Δ*popZ_Mgr_* cells. (Left image) DIC. Minicells are indicated by white arrowheads. (Right image) DAPI channel maximum-intensity projection of a deconvolved z-stack. The calibration bar indicates the intensity of fluorescence. Scale bar = 3 µm. (B) CMCF (corrected mean cell fluorescence) determined from nondeconvolved DAPI channel images. Bar charts are given as mean + SD (*n* = 19 cells each). *P* values were determined as described in the legend to Fig. 2.

Since one of the major functions of PopZ in both C. crescentus and A. tumefaciens is also in regulation of chromosome segregation (17, 18, 24), we quantified DNA content of Δ*popZ_Mgr_* minicells by staining with DAPI (4′,6-diamidino-2-phenylindole), a dye specific for DNA (Fig. 4A). Remarkably, minicells had an approximately 2.8-fold or 1.5-fold reduced mean cell fluorescence (Fig. 4B) compared to either other cells or their polar regions (to account for possible volume differences of minicells, since cells get thinner toward the poles), further suggesting that PopZ in M. gryphiswaldense also contributes to proper chromosome segregation.

### Deletion of *popZ_Mgr_* impairs motility and magneto-aerotaxis.

**FIG 5:**
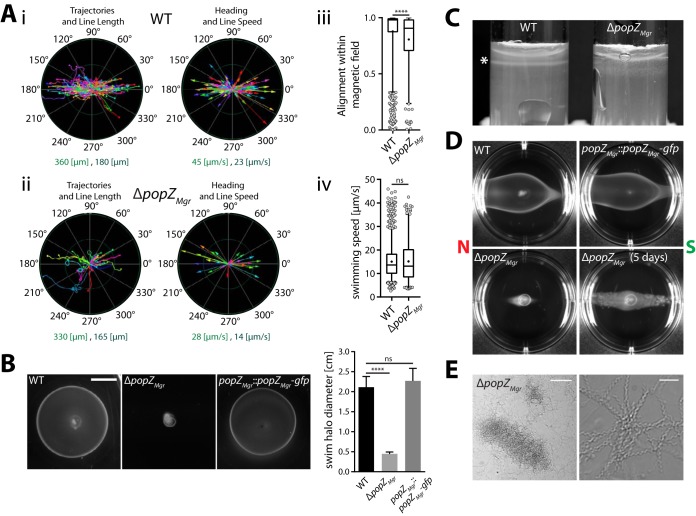
Characterization of Δ*popZ_Mgr_* cell motility and magneto-aerotactic behavior. (Ai and ii) Representative tracking results from magnetic nonpolar wild type (Ai; *C*_mag_ = 1.21, *n* = 400 tracks) and Δ*popZ_Mgr_* (Aii; *C*_mag_ = 0.91, *n* = 49 tracks) cells in a 500-µT magnetic field determined at 24°C. “Swimming trajectories” (trajectory length corresponds to the appropriate track length) and “heading” (depicted as colored arrow, where arrow length corresponds to the appropriate object speed) are plotted in a polar coordinate system. The ranges for “line length” and “line speed” (indicated by the inner ring [dark green] and outer ring [light green] of the polar coordinate system) are given below the graphs. Magnetic north corresponds to 0° and magnetic south to 180°, respectively. Note the Δ*popZ_Mgr_* population consisted of small cells that traveled long distances and displayed high swimming speeds, but were not very well aligned within the magnetic field, and slow-swimming elongated cells, which were well aligned. (Aiii and iv) Overall alignment and swimming speed distributions within a 500-µT magnetic field for the wild-type and Δ*popZ_Mgr_* strains estimated by motility tracking. Results were pooled from data sets of at least 3 independent experiments (performed on different days), resulting in total numbers of 1,115 tracks for the wild type (mean *C*_mag_ = 1.2 ± 0.07) and 243 tracks for the Δ*popZ_Mgr_* strain (mean *C*_mag_ = 0.94 ± 0.03). Box plots are as described in legend to Fig. 2. *P* values were determined by Mann-Whitney U test (****, *P* < 0.0001; ns, not significant [*P* ≥ 0.05]). (B) Aerotactic swim halo formation of M. gryphiswaldense wild-type, Δ*popZ_Mgr_*, and *popZ_Mgr_*::*popZ_Mgr_*-*gfp* strains in 0.2% motility agar 3 days after inoculation and incubation at 28°C and 2% atmospheric oxygen (scale bar = 1 cm). Deletion of *popZ_Mgr_* almost completely abolished aerotactic swim ring formation, whereas integration of *gfp* at the chromosomal *popZ_Mgr_* locus did not negatively affect motility. Swim ring diameters were estimated from at least three independent experiments. Values are given as mean + SD. Statistical analysis in panel B was performed as described in the legend to Fig. 2. (C) Aerotactic band formation (marked with an asterisk) in 0.3% soft agar tubes is impaired in the Δ*popZ_Mgr_* strain. (D) Swim ring assay of polar wild-type, Δ*popZ_Mgr_*, and *popZ_Mgr_*::*popZ_Mgr_*-*gfp* strains in the presence of a magnetic field. Cells were grown for several passages under conditions permissive to enrich cells with a south-seeking (SS) swimming polarity bias before the experiment. Six-well plates with 0.2% motility agar were incubated between two strong permanent magnets (∼100 mT) for 2 days at 28°C under atmospheric conditions (20% oxygen). Growth-dependent migration along the magnetic field for the Δ*popZ_Mgr_* strain was only observed after additional 3 days of incubation (indicated in the figure with a total incubation time of 5 days). Polar cultures (SS) of the wild-type and *popZ_Mgr_*::*popZ_Mgr_*-*gfp* strains exhibited a biased movement toward the northern magnetic pole (south seeking), which was confirmed by the hanging drop assay (not shown), whereas polar behavior of the Δ*popZ_Mgr_* strain was apparently lost. The direction of the magnetic field is indicated in the figure. Similar results were obtained with a coil setup (Fig. S4B and C), which provides a weaker and more uniform magnetic field. (E) Cell filamentation of the Δ*popZ_Mgr_* strain raises the tendency of flocculation and cell clumping. Pictures show magnifications of cell aggregates. Scale bars correspond to 100 µm (left picture) and 10 µm (right picture), respectively.

In M. gryphiswaldense, magnetotaxis is tightly coupled to aerotaxis in order to govern directed swimming toward optimal low-oxygen levels (13, 32). Remarkably, it was also found that cells that perform polar magneto-aerotaxis and display a distinct swimming polarity bias within the magnetic field (preferentially north or south seeking) can be enriched within only few generations (13). Thus, during cytokinesis the proper segregation of magnetosome chains with an inherent physically imprinted magnetic polarity must be coordinated with the determination of the magnetotactic swimming direction. In order to analyze if the Δ*popZ_Mgr_* strain is affected with respect to motility and magneto-aerotaxis, we performed tracking microscopy (Fig. 5A) and various soft-agar-based assays (Fig. 5B to D). Most cells of the Δ*popZ_Mgr_* strain contained bipolar flagella as in the wild type (Fig. 2Aiii and 2iv), but occasionally additional flagella were observed along the cell body (Fig. 2Aii). Elongated cells of the *popZ_Mgr_* deletion strain were motile and despite their highly increased length still aligned to an external magnetic field when observed by dark-field microscopy (Fig. 5Aii; see Movie S3 in the supplemental material) or optical measurement of their magnetic response (*C*_mag_). In general, the Δ*popZ_Mgr_* population was heterogeneous, consisting of smaller fast-swimming cells and more elongated cells that moved at a lower speed than the wild type (Movie S3 [movies of the wild-type strain can be found in reference 13]). Shorter cells, which had higher swimming speeds and traveled longer distances within the time frame of observation, were observed to swim in circular motions and were not well aligned within the magnetic field (Fig. 5Aii and Movie S3), which might be due to their altered cell length or the existence of no or only short magnetosome chains. In contrast, longer cells, which displayed low swimming speeds and traveled only short distances, were very well aligned within the magnetic field, presumably due to their overly elongated magnetosome chains. In summary, due to the higher number of highly motile short cells, the mean overall alignment of the Δ*popZ_Mgr_* population was reduced compared to that of the wild type (Fig. 5Aiii), whereas the swimming speed distribution did not differ significantly from the wild type (Fig. 5Aiv). Accordingly, Δ*popZ_Mgr_* cultures also reproducibly displayed a slightly lower *C*_mag_ than the wild type (as determined from at least triplicate cultures [values are given in the legend to Fig. 5Aiii and Aiv]). In contrast to the wild type, the formation of aerotactic swim halos in semisolid medium was almost completely abolished (Fig. 5B). Moreover, in comparison to the wild type, which forms sharp aerotactic bands in soft agar tubes, the Δ*popZ_Mgr_* mutant grew only in a diffuse zone close to the surface (Fig. 5C). When soft agar assays were performed in the presence of a magnetic field (Fig. 5D; see Fig. S4C in the supplemental material), spreading of the *popZ_Mgr_* deletion mutant parallel to the magnetic field was only observed after prolonged incubation (>4 days), confirming that cells are still able to align and distribute along the magnetic field lines, but in a slow and possibly only growth-dependent manner. We also failed to restore a swimming polarity bias in elongated Δ*popZ_Mgr_* cells by magnetic selection. Whereas the wild-type and *popZ_Mgr_*::*popZ_Mgr_-gfp* strains displayed a south-seeking polarity bias upon repeated passaging in O_2_ gradients within a superimposed vertical Southern Hemisphere-like magnetic field, Δ*popZ_Mgr_* cells were rather equally distributed toward both magnetic poles (Fig. 5D and Fig. S4C). These findings were confirmed by the hanging drop assay (not shown). In general, only few cells of the Δ*popZ_Mgr_* strain accumulated in equal proportions at the northern and southern magnetic pole (facing the air-adjacent borders of the drop), speaking for a general impairment of aerotaxis. In summary, deletion of *popZ_Mgr_* severely impaired motility and aerotaxis. Since we observed an increased tendency of highly elongated Δ*popZ_Mgr_* cells to intertwine and aggregate (Fig. 5E), reduced motility in soft agar is also partially explained by the formation of cell clumps.

10.1128/mBio.02716-18.4 FIG S4 Custom magnetic equipment used for motility analysis and swim halo assay of the wild-type, Δ*popZ_Mgr_*, and *popZ_Mgr_*::*popZ_Mgr_-gfp* strains in the presence of a homogenous magnetic field. (A) Custom microscope setup for motility tracking equipped with a pair of magnetic coils. (B) Coil setup used for growth of cells under conditions permissive to enrich cells with swimming polarity bias before the soft agar experiment. Therefore, cells were repeatedly passaged in culture tubes that were incubated in an ∼0.6-mT Southern Hemisphere-like magnetic field applied in the z direction. (C) Upon repeated passages in a Southern Hemisphere-like magnetic field, cultures were used for inoculation of 0.2% motility agar in 6-well plates, following incubation using the same setup with an ∼0.6-mT magnetic field applied in the y direction for 2 days at 28°C under atmospheric conditions (or an additional 3 days of incubation for the Δ*popZ_Mgr_* mutant, indicated with an incubation time of 5 days). The direction of the magnetic field is indicated in the figure with an arrow (pointing from magnetic north to south). Download FIG S4, JPG file, 2.7 MB. Copyright © 2019 Pfeiffer et al. 2019 Pfeiffer et al. This content is distributed under the terms of the Creative Commons Attribution 4.0 International license.

10.1128/mBio.02716-18.9 MOVIE S3 Video microscopy of swimming cells of the Δ*popZ_Mgr_* strain. Dark-field video microscopy (200× magnification) was recorded at 25 fps and 24°C in a homogenous magnetic field of 400 µT. The direction of the magnetic field is indicated with an arrow (pointing from magnetic north to south). Download Movie S3, MPG file, 1.5 MB. Copyright © 2019 Pfeiffer et al. 2019 Pfeiffer et al. This content is distributed under the terms of the Creative Commons Attribution 4.0 International license.

### MamK filament dynamics is independent of PopZ*_Mgr_*.

In M. gryphiswaldense, magnetosome chains are recruited to midcell to ensure equal partitioning of magnetosomes to both daughter cells. It has been recently found that the MamK filament has a particular dynamic behavior, growing from both cell poles, elongating toward midcell, and undergoing treadmilling (7).

**FIG 6:**
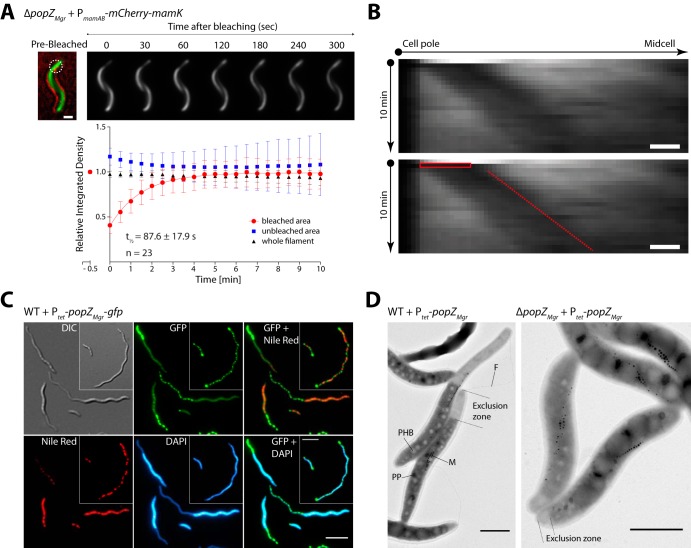
Photokinetic analysis of MamK filament dynamics upon *popZ_Mgr_* absence and effects on cell morphology upon overexpression of *popZ_Mgr_*. (A) Photobleaching of mCherry-MamK was used to follow the recovery of the fluorescence corresponding to the MamK filament during 10 min in the Δ*popZ_Mgr_* strain. The mCherry-MamK translational fusion was expressed from a replicative plasmid under the control of the native P*_mamAB_* promoter. The upper panel shows representative cells for this assay, indicating the selected bleached areas (white dashed circle) and fluorescence recovery progression over time. The prebleaching image is a composite of the bright-field and fluorescence channels to display subcellular localization. The lower panel shows the quantification of the MamK filament fluorescence recovery over time. Time point zero was measured immediately after the laser pulse. The half-time fluorescence recovery is presented as *t*_1/2_ in the plot. Scale bar = 1 μm. (B) MamK filament pole-to-midcell treadmilling growth behavior analysis by FRAP in the Δ*popZ_Mgr_* strain. Kymographs display the fluorescence signal intensity (*x* axis) of bleached mCherry-MamK filaments over time (*y* axis). The corresponding duplicated kymograph indicates bleaching time/area (red box) and filament fluorescent signal progression (red dashed line). The bleach-marked filaments were followed for 10 min (imaging every 30 s). Scale bars = 500 nm. (C) Prolonged overexpression (23 h postinduction) of *popZ_Mgr_*-*gfp* results in polar regions devoid of chromosomal DNA (stained with DAPI) and PHB granules (Nile red stain). The inset shows an additional cell with multiple PopZ foci distributed across the cell body. Scale bars = 5 µm. (D) Upon overproduction of PopZ*_Mgr_* (25 h postinduction), a polar exclusion zone devoid of ribosomes, polyhydroxybutyrate (PHB), and polyphosphate (PP) granules is formed. Formation of a large PopZ exclusion zone does not prevent formation of flagella (F) at the same pole. In some cells, the magnetosome chains (M) were found to be enclosed in the PopZ-rich zone. Scale bars = 1 µm.

Therefore, based on the PopZ*_Mgr_* localization pattern, we asked whether PopZ*_Mgr_* is involved in or influences the MamK dynamics. To examine this hypothesis, we performed photokinetic analysis of the MamK filament in Δ*popZ_Mgr_* cells. Fluorescence recovery after photobleaching (FRAP) of MamK filaments using an mCherry-MamK fusion showed a half-time fluorescence recovery (*t*_1/2_) of 87.6 ± 17.9 s (Fig. 6A). Recently, it was reported that the mCherry-MamK translational fusion expressed in M. gryphiswaldense wild-type cells from a plasmid and chromosomally showed *t*_1/2_ values of 71.8 ± 6.6 and 68.3 ± 4.8 s, respectively (7). A one-way analysis of variance (ANOVA) followed by a Tukey's multiple-comparison test determined that the mCherry-MamK filament *t*_1/2_ in the absence of *popZ_Mgr_* is statistically not significant compared to the previously reported values for the wild-type strain (*P* < 0.05). Furthermore, the MamK pole-to-midcell growth and its treadmilling behavior are not affected upon absence of *popZ_Mgr_* (Fig. 6B). Thus, it can be concluded that the MamK filament dynamics, especially the directed pole-to-midcell growth, is independent of PopZ*_Mgr_*.

### PopZ*_Mgr_* forms a polar exclusion zone devoid of macromolecules and chromosomal DNA.

To study the effect of *popZ_Mgr_* overexpression, PopZ*_Mgr_* and PopZ*_Mgr_*-GFP were overproduced in *trans* under the control of P*_tet_* (anhydrotetracycline-inducible promoter) in the M. gryphiswaldense wild type and Δ*popZ_Mgr_* strain. Upon reintroduction of *popZ_Mgr_* (or *popZ_Mgr_-gfp*), cell morphology of the wild type, formation of swim halos, and growth were restored in the Δ*popZ_Mgr_* strain (see Fig. S5 in the supplemental material). Furthermore, prolonged overexpression of *popZ_Mgr_* or *popZ_Mgr_-gfp* in the wild-type strain caused severe cell filamentation and delayed growth, whereas expression in the Δ*popZ_Mgr_* strain had a lesser effect on growth and cell length, likely due to the absence of endogenous PopZ*_Mgr_* (Fig. 6D and Fig. S5). Moreover, PopZ*_Mgr_*-GFP overproduction in the M. gryphiswaldense wild type caused either (i) cells with two large polar foci and multiple smaller foci distributed across the cell and in between the PHB granules (stained with the lipophilic dye Nile red, specific for membranes and polyhydroxybutyrate [PHB]) (Fig. 6C, inset) or (ii) cells with a large PopZ accumulation cluster expanding from one pole (Fig. 6C). In the latter, some cells had an additional smaller PopZ*_Mgr_*-GFP cluster at the opposite pole. The PopZ expansion zone encompassed several micrometers in length and presented a reduced cell diameter, resulting in a tail-like appearance. Additional staining with DAPI and Nile red revealed that chromosomal DNA and PHB granules were excluded from the expanded PopZ area. Transmission electron microscopy (TEM) analysis confirmed that this zone was depleted of larger cytoplasmic structures such as PHB or polyphosphate granules (Fig. 6D). Furthermore, the brighter appearance indicated that the putative polar PopZ-rich region is mostly devoid of electron-dense cytoplasmic structures and macromolecules (e.g., ribosomes). Even upon PopZ*_Mgr_* overexpression, magnetosome chains were still located at midcell, resembling the wild-type phenotype, but in a few cases were also embedded into the outermost part of the PopZ expansion zone. Of note, the formation of flagella at the PopZ-rich poles was not impaired (Fig. 6D). In summary, PopZ*_Mgr_* forms a polar expansion zone that is depleted in larger macromolecules and organelles, similar to previously reported observations regarding PopZ in C. crescentus (15, 17).

10.1128/mBio.02716-18.5 FIG S5 Transcomplementation of Δ*popZ_Mgr_* and overexpression of *popZ_Mgr_* or *popZ_Mgr_-gfp*. (A) Representative images taken from time series and induction experiments with the M. gryphiswaldense wild-type and Δ*popZ_Mgr_* strains harboring random single-copy chromosomal insertions of a P*_tet_*-*popZ_Mgr_* or -*popZ_Mgr_-gfp* expression cassette. Micrographs are shown for different time points following postinduction with 50 ng/ml anhydrotetracycline. Expression of *popZ_Mgr_* or *popZ_Mgr_-gfp* was sufficient to transcomplement the cell filamentation phenotype of the Δ*popZ_Mgr_* strain. Some insertion mutants displayed cell shortening even before anhydrotetracycline induction—possibly caused by a growth advantage for transposon insertion mutants harboring insertion sites or mutations that favor increased or leaky expression levels of *popZ_Mgr_*/*popZ_Mgr_-gfp* from the P*_tet_* promoter system or promoters encoded upstream of the random integration site. Prolonged overproduction of PopZ*_Mgr_* or PopZ*_Mgr_*-GFP in the wild-type background caused severe cell filamentation and (B) formation of a DNA exclusion zone visible by DAPI staining (marked with an arrowhead). Scale bars correspond to 5 µm for all fluorescent images. (C) Reintroduction of *popZ_Mgr_* or *popZ_Mgr_-gfp* in the Δ*popZ_Mgr_* strain restores aerotactic swim halo formation in semisolid medium (scale bar = 1 cm). Cells grown in liquid medium (6 h postinduction with 50 ng/ml of anhydrotetracycline) were inoculated into 0.2% motility agar, and plates were incubated for 3 days at 28°C under atmospheric conditions. Download FIG S5, JPG file, 2.8 MB. Copyright © 2019 Pfeiffer et al. 2019 Pfeiffer et al. This content is distributed under the terms of the Creative Commons Attribution 4.0 International license.

### Bipolar PopZ*_Mgr_* localization requires host-specific factors.

**FIG 7:**
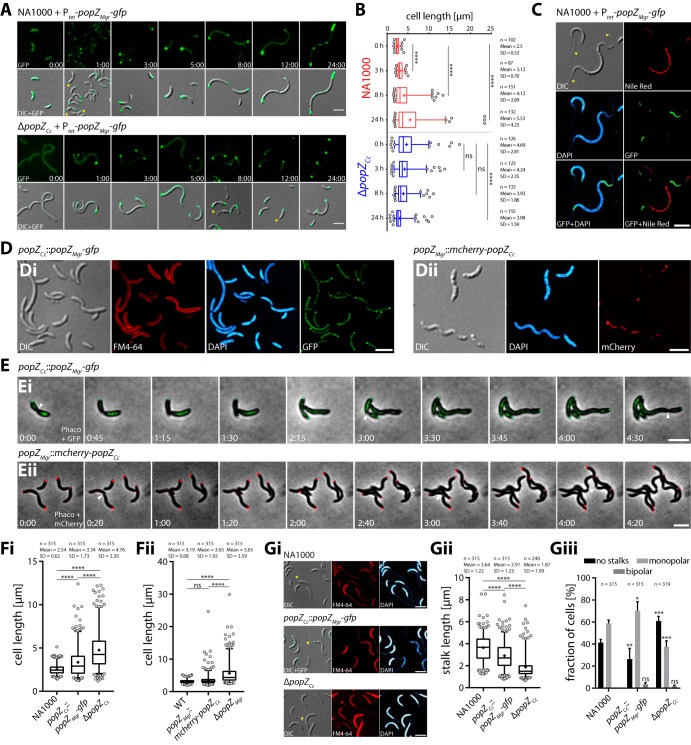
PopZ*_Mgr_* and PopZ*_Cc_* are able to partially replace their reciprocal functions. (A) Heterologous expression of *popZ_Mgr_*-*gfp* in *trans* from P*_tet_* in the presence (C. crescentus NA1000 wild type) and absence (Δ*popZ_Cc_* strain) of endogenous *popZ_Cc_*. Numbers indicate hours and minutes postinduction with 50 ng/ml anhydrotetracycline during growth in PYE (peptone-yeast extract) medium. Overproduction of PopZ*_Mgr_*-GFP in NA1000 (5- to 24-h time points) leads to formation of polar PopZ-rich regions and cell filamentation, whereas expression in the Δ*popZ_Cc_* strain causes shortening of cells. The first row shows the fluorescence channel, and the second row shows an overlay image of DIC and the fluorescence channel. (B) Cell length distributions at selected time points for the two strains shown in panel A. (C) C. crescentus NA1000 cells expressing *popZ_Mgr_*-*gfp* (23 h postinduction with 50 ng/ml anhydrotetracycline). Since only a few PHB granules were formed in C. crescentus during cultivation in rich PYE medium, the cells shown were cultivated in HIGG (Hutner base–imidazole-buffered–glucose–glutamate) minimal medium with limiting amounts of phosphate (0.01 mM), which did induce formation of large amounts of PHB storage granules in addition to stalk elongation. Note PopZ*_Mgr_* forms large polar exclusion zones devoid of DNA and PHB granules. Furthermore, overexpression of PopZ*_Mgr_*-GFP did cause cell elongation and affected stalk formation (cells with bipolar stalks [yellow arrowheads]). Cells were stained with DAPI and Nile red. (Di) Localization of PopZ*_Mgr_*-GFP in C. crescentus when expressed as the sole source of PopZ from the endogenous *popZ_Cc_* promoter (*popZ_Cc_*::*popZ_Mgr_*-*gfp* strain). Cells were grown in PYE medium. (Dii) Localization of mCherry-PopZ*_Cc_* in M. gryphiswaldense when expressed as sole source of PopZ from the endogenous *popZ_Mgr_* promoter (*popZ_Mgr_*::*mCherry-popZ_Cc_* strain). Cells were grown in flask standard medium (FSM). (E) Time-lapse microscopy of strains shown in panel D. Numbers indicate hours and minutes. Note both PopZ orthologs display delayed or erratic polar localization when expressed in the opposing parent strain. (F) Analysis of cell length of C. crescentus (Fi) and M. gryphiswaldense (Fii) strains harboring a chromosomal site-specific replacement of their PopZ ortholog compared to the respective wild-type and Δ*popZ* strains. C. crescentus strains were grown in PYE to an OD_600_ of ∼0.17. M. gryphiswaldense strains were grown under microaerobic conditions to an OD_565_ of ∼0.2 in FSM medium. Note both PopZ orthologs are capable to partially replace their reciprocal functions with respect to the cell-length phenotype compared to strains harboring *popZ* deletions. (G) The C. crescentus wild-type NA1000, Δ*popZ_Cc_*, and *popZ_Cc_*::*popZ_Mgr_*-*gfp* strains were grown in HIGG minimal medium containing 0.1 mM phosphate to an OD_600_ of ∼1.5 for the analysis of stalks. (Gi) Epifluorescence micrographs of all three strains stained with FM4-64 and DAPI. Note since FM4-64 did not reliably stain stalks of all cells, DIC microscopy and DAPI staining (which unspecifically stains stalks) were included to identify and measure stalks. (Gii and Giii) Analysis of (Gii) stalk length and (Giii) stalk number (given as percentage of cells with no stalks or mono- or bipolar stalks). Note PopZ*_Mgr_* is able to partially complement the function of PopZ*_Cc_* with respect to proper stalk formation. Measurements shown in panel B were taken from one representative induction experiment. Experiments shown in panels F and G were performed in biological triplicates (independent cultures). The total number of analyzed cells (*n*) is indicated in the graphs. Box plots and statistical analysis are similar as described in the legend to Fig. 2. Epifluorescence micrographs shown in panels C to E and G are maximum-intensity projections of deconvolved z-stacks. Fluorescence channels are indicated in the graph. Representative stalks and cell division events are exemplarily marked with yellow and white arrowheads, respectively. All scale bars = 3 µm.

In C. crescentus, PopZ (abbreviated PopZ*_Cc_*) first localizes to the old pole and undergoes a transition from monopolar to bipolar after completion of cell division (17). The C. crescentus life cycle is highly asymmetric, generating a smaller and motile swarmer cell and a stalked cell that possesses a tubular extension at the old pole, required for surface attachment. The distinct bipolar localization pattern in M. gryphiswaldense (Fig. 1) prompted us to investigate the localization pattern of PopZ*_Mgr_* in C. crescentus (Fig. 7; see Fig. S6A in the supplemental material). When PopZ*_Mgr_*-GFP was heterologously produced in C. crescentus NA1000 (in *trans* expressed from P*_tet_* in the presence of endogenous PopZ*_Cc_*), a unipolar-to-bipolar transition pattern was revealed (Fig. 7A, 1-h time-point and Fig. S6A), similar to the localization pattern of PopZ*_Cc_*. PopZ*_Mgr_* and PopZ*_Cc_* are conserved in their N- and C-terminal regions (37.2% identity and 51.3% similarity, in a global alignment, including some of the most related orthologs [Fig. S1B to D]), which are known to be important in C. crescentus for interaction with the ParA/ParB chromosome segregation machinery and PopZ cluster formation, respectively. Thus, the observed localization pattern of PopZ*_Mgr_* in C. crescentus might be explained by a direct interaction between PopZ*_Mgr_* and PopZ*_Cc_* and/or with other known PopZ interactors present in C. crescentus, such as ParA/ParB. Upon prolonged expression of *popZ_Mgr_-gfp* in C. crescentus, cells became heavily elongated and aberrantly shaped (Fig. 7A and B), indicating that overexpression of PopZ*_Mgr_* also interferes with cell division in C. crescentus. In addition, heterologous *popZ_Mgr_-gfp* overproduction in C. crescentus resulted in the appearance of multiple PopZ foci and large polar PopZ exclusion zones (Fig. 7A), similar to the previously described observations regarding the overproduction of native PopZ*_Cc_* in C. crescentus (15, 17). As observed for M. gryphiswaldense (Fig. 6C and D), PopZ*_Mgr_* exclusion zones in C. crescentus were devoid of DNA and PHB storage granules (Fig. 7C). Heterologous expression of *popZ_Mgr_-gfp* in the Δ*popZ_Cc_* background partially restored the cellular morphology of the C. crescentus wild-type strain (Fig. 7A), resulting in a reduced cell length close to wild-type-like levels (Fig. 7B). However, in some cells, we observed PopZ*_Mgr_*-GFP foci located at ectopic positions opposite to the stalked pole (Fig. 7A, 12-h time point, yellow arrowheads), indicating that the absence of PopZ*_Cc_* was not fully transcomplemented by PopZ*_Mgr_*-GFP.

10.1128/mBio.02716-18.6 FIG S6 Localization of PopZ*_Mgr_* in C. crescentus and various other bacterial hosts (E. coli, R. sphaeroides, and R. rubrum). (A) Localization of PopZ*_Mgr_*-GFP in C. crescentus NA1000 cells stained with FM4-64 and DAPI (for the analysis of stalks, in addition to the DIC channel) 1 h postinduction with 50 ng/ml anhydrotetracycline. The corresponding graphs show a mean line plot of fluorescence intensity of the DAPI and GFP channel (*n* = 39 cells), and a demograph of the GFP channel that depicts the cell-cycle-dependent localization pattern (*n* = 260 cells). Fluorescence profiles were sorted with the stalked pole oriented to the left. Note PopZ*_Mgr_* localizes in a unipolar-to-bipolar transition pattern in C. crescentus. (B) Localization of PopZ*_Mgr_*-GFP in E. coli WM3064 cells stained with DAPI 2 h postinduction with 50 ng/ml anhydrotetracycline. PopZ_*Mgr*_ exhibits a monopolar localization pattern in DNA-free regions and localizes randomly to either the new or old pole. Diagrams show a mean line plot of fluorescence intensity for the DAPI and GFP channel of *n* = 94 cells. The fluorescent pole was oriented to the left. (C) Localization of PopZ*_Mgr_*-GFP in E. coli WM3064 spheroblasts 1 h postinduction with 50 ng/ml anhydrotetracycline in the absence of DAP. Cells were washed four times to remove residual DAP and then transferred in DAP-free LB medium. PopZ foci appear at random positions close to the cell boundary. (D) Localization of PopZ_*Mgr*_-GFP in R. sphaeroides and (E) R. rubrum. Cells were stained with DAPI. Note PopZ*_Mgr_* localizes to DNA-free polar regions. In contrast to the monopolar localization observed in cells of R. sphaeroides, PopZ*_Mgr_* generally localized to both poles and midcell in dividing cells (white arrowhead) in R. rubrum. Shown are representative images from time series 4 h postinduction with 50 ng/ml anhydrotetracycline. The respective line plots for the DAPI and GFP channel are given for *n* = 244 cells for R. sphaeroides (fluorescent pole oriented to the left) and *n* = 328 cells for R. rubrum (random sorting of fluorescent profiles). In addition, two demographs of the GFP plus DAPI channel depict the cell-cycle-dependent localization of PopZ*_Mgr_*-GFP in R. rubrum. (Line plots and demographs) Fluorescence profiles were determined from nondeconvolved micrographs as described in detail in Materials and Methods. To generate line plots, fluorescence profiles were sorted evenly into three groups of short (red), medium (blue), and long (green) cells. (Fluorescence micrographs) The epifluorescence micrographs shown in panels A and E are maximum-intensity projections of deconvolved z-stacks or deconvolved single-plane images in panels B, C, and D. Fluorescence channels are indicated in the graph. Representative stalks and cell division events are exemplarily marked with yellow and white arrowheads, respectively. All scale bars = 3 µm. Download FIG S6, JPG file, 2.8 MB. Copyright © 2019 Pfeiffer et al. 2019 Pfeiffer et al. This content is distributed under the terms of the Creative Commons Attribution 4.0 International license.

In order to avoid artifacts caused by altered expression levels (due to expression from a random ectopic locus under the control of P*_tet_* or the presence of endogenous PopZ*_Cc_*), we constructed a C. crescentus strain harboring a site-specific chromosomal replacement of *popZ_Cc_* against *popZ_Mgr_*-*gfp*. We also performed the reciprocal experiment and constructed an M. gryphiswaldense strain that carries a site-specific chromosomal replacement of *popZ_Mgr_* for an *mCherry*-*popZ_Cc_* fusion. Notably, PopZ*_Mgr_*-GFP localized in a monopolar-to-bipolar fashion in C. crescentus (Fig. 7Di and Ei), when expressed from the endogenous *popZ_Cc_*-promoter as the sole source of PopZ present. However, transition of PopZ*_Mgr_*-GFP to the new poles occurred with ectopic timings, and not all PopZ*_Mgr_* was retained in polar regions, as indicated by the diffuse cytoplasmic fluorescence signal (Fig. 7Di and Ei). In M. gryphiswaldense, mCherry-PopZ*_Cc_* in general did localize to both cell poles (Fig. 7Dii and Eii). However, one cell pole displayed much stronger fluorescence, and filamentous localization patterns were observed (Fig. 7Dii, elongated cell at the bottom left). Furthermore, the appearance of PopZ foci at the new cell poles was delayed, and disappearance of polar foci was observed in some cases (Fig. 7Eii). Together, these results indicated that bipolar PopZ localization is regulated by host-specific proteins in M. gryphiswaldense and that both PopZ orthologs differ to an extent that does not allow full functionality within the cell cycle of the heterologous host. The findings that bipolar PopZ subcellular localization is not inherent to the protein itself but rather host specific were further corroborated by expression of PopZ*_Mgr_*-GFP in various other bacterial hosts (as shown in Fig. S6B to E), including only distantly related Escherichia coli and the two other alphaproteobacteria Rhodobacter sphaeroides and Rhodospirillum rubrum, which contain no PopZ (R. sphaeroides) or an endogenous PopZ ortholog (R. rubrum [49% identical to PopZ*_Mgr_*]) (28). Expression of PopZ*_Mgr_*-GFP in E. coli WM3064 resulted in the formation of large fluorescent clusters in polar nucleoid-free regions, exhibiting unipolar localization at either the new or old pole (Fig. S6B), similar to the observations made upon expression of PopZ*_Cc_* in E. coli (17, 18). When we studied the localization of PopZ*_Mgr_*-GFP in spheroid 2,6-diaminopimelic acid (DAP)-auxotrophic WM3064 cells formed after depletion of DAP, random foci were formed close to the cell periphery (Fig. S6C), indicating that localization of PopZ_*Mgr*_ does not depend on geometrical constraints. In ovoid rod-shaped R. sphaeroides cells, PopZ*_Mgr_* generally localized only at the old pole (Fig. S6D). A similar bipolar pattern to that in M. gryphiswaldense was observed in spirillum-shaped *R. rubrum* cells, with two new PopZ foci emerging at the site of cell division (Fig. S6E). In summary, these results suggest that monopolar accumulation in DNA-free polar regions occurs by a mechanism that is inherent to PopZ*_Mgr_*, whereas bipolar localization apparently depends on distinct alphaproteobacterium-specific host factors.

To further investigate whether PopZ orthologs can replace their functionalities, we compared median cell lengths of M. gryphiswaldense and C. crescentus strains harboring reciprocal PopZ orthologs with the respective wild-type and Δ*popZ* strains. The median cell length of the C. crescentus *popZ_Cc_*::*popZ_Mgr_*-*gfp* strain was 1.5-fold reduced compared to the Δ*popZ_Cc_* strain (and 1.2-fold higher than that of the NA1000 wild-type strain [Fig. 7Fi]), whereas the median cell length of the M. gryphiswaldense *popZ_Mgr_*::*mCherry*-*popZ_Cc_* strain was 1.4-fold lower than that of the Δ*popZ_Mgr_* strain (but only 1.05-fold larger than that of the M. gryphiswaldense wild-type strain [Fig. 7Fii]). These results indicated that in both strains, the loss of the respective PopZ ortholog can be partially rescued by expression of the reciprocal ortholog.

To analyze whether PopZ*_Mgr_* is capable to accomplish functions with respect to stalk formation, which is impaired in the C. crescentus *popZ* deletion strain (17, 18), we also analyzed stalk length and frequency in cells grown under phosphate-limiting conditions, known to cause severe stalk elongation (33), to facilitate detection and analysis of stalks (Fig. 7G). In contrast to previous reports that the Δ*popZ_Cc_* strain does not form stalks (3, 21), we found that the Δ*popZ_Cc_* strain grown under phosphate starvation is still able to form stalks, but of 2.5-fold-reduced median length (Fig. 7Gii) and at a lower frequency (Fig. 7Giii) than the NA1000 wild-type strain. In comparison, the fraction of cells with monopolar stalks was 1.6-fold lower in the Δ*popZ_Cc_* mutant than in the NA1000 wild-type strain. In contrast, the fraction of cells without stalks was 2.3-fold reduced in the *popZ_Cc_*::*popZ_Mgr_*-*gfp* strain compared to the Δ*popZ_Cc_* strain, whereas the number of cells with monopolar stalks was 1.9-fold higher, bringing both values closer to wild-type levels. A minor fraction of cells with bipolar stalks was detected within the Δ*popZ_Cc_* and *popZ_Cc_*::*popZ_Mgr_*-*gfp* populations, whereas no cells containing bipolar stalks were found in the NA1000 wild type. Median stalk lengths of the *popZ_Cc_*::*popZ_Mgr_*-*gfp* strain were still 1.4-fold lower relative to the wild type but 1.8-fold larger in comparison to the Δ*popZ_Cc_* strain. Notably, stalk formation was also restored upon expression of *popZ_Mgr_*-*gfp* in the Δ*popZ_Cc_* strain from P*_tet_* (Fig. 7A, 8 and 12 h, yellow arrowheads), and overexpression of PopZ*_Mgr_*-GFP in NA1000 caused aberrant bipolar stalk formation (Fig. 7C, yellow arrowheads). In summary, these results argue that PopZ*_Mgr_* is able to partially accomplish functions inherent to PopZ*_Cc_* with respect to stalk formation in C. crescentus.

## DISCUSSION

In C. crescentus, PopZ has been described as an important landmark protein, generating a polar hub domain for multiple proteins involved in cell cycle control and polar morphogenesis (14–18, 34, 35). In addition to C. crescentus, PopZ has been studied in A. tumefaciens (23–26), which exhibits unipolar growth by addition of peptidoglycan at the new “growth pole” (22). Here, we report that PopZ in the magnetotactic model organism M. gryphiswaldense plays a similar, but somewhat distinct role. In contrast to C. crescentus and A. tumefaciens, where cell division results in morphologically distinct cells and/or daughter cells that differ in cell cycle progression, division in M. gryphiswaldense gives rise to morphologically nearly equal daughter cells. Deletion and overexpression of *popZ* in M. gryphiswaldense resulted in severe cell division defects (Fig. 2, Fig. 3, Fig. S3, Movie S1, Movie S2, and Fig. 6C and D and Fig. S5A and B, respectively) and DNA missegregation (Fig. 4), consistent with previous observations in C. crescentus (17, 18) and A. tumefaciens (24, 25). However, we did not observe formation of ectopic poles and cell branching as in A. tumefaciens (25, 26). In accordance with reported results in C. crescentus (15, 17), we have observed formation of large exclusion zones upon overproduction of PopZ*_Mgr_* in M. gryphiswaldense (Fig. 6C and D). These results imply that PopZ*_Mgr_* may have an important role as a putative landmark protein and in the control of cell-cycle-related factors in M. gryphiswaldense. As for now, it can only be speculated that the severe cell elongation and minicell formation of the Δ*popZ_Mgr_* strain are due to an indirect impairment in FtsZ ring positioning. In C. crescentus, MipZ inhibits FtsZ polymerization by generating a gradient with the highest concentration in polar regions via ParB-PopZ-dependent retention of MipZ (19, 20), thus creating a region with the lowest MipZ concentration at midcell with suitable conditions for FtsZ ring positioning and formation. Since orthologs of the ParA/ParB chromosome segregation system and MipZ spatial regulator are present in M. gryphiswaldense, it is likely that PopZ*_Mgr_* contributes to stabilization of the MipZ gradient and, thereby, proper placement of the division site. However, the specific functions of ParA, ParB, and MipZ in M. gryphiswaldense remain to be elucidated.

10.1128/mBio.02716-18.8 MOVIE S2 Cryo-electron tomography and three-dimensional rendering of a filamentous Δ*popZ_Mgr_* cell. The view is through the z-stack tomographic slices of two tomograms, the cell pole and cell body areas from the same elongated Δ*popZ_Mgr_* cell, at a 22,500× magnification. Cellular envelope is blue, chemoreceptor arrays are purple, magnetosome vesicles are yellow, magnetite is red, and the MamK filament is green. This concatenated movie is related to Fig. 3. Download Movie S2, MPG file, 18.1 MB. Copyright © 2019 Pfeiffer et al. 2019 Pfeiffer et al. This content is distributed under the terms of the Creative Commons Attribution 4.0 International license.

Deletion of *popZ* in M. gryphiswaldense severely affected motility and apparently polar magneto-aerotactic behavior (Fig. 5, Fig. S4, and Movie S3). Inheritance of a specific magnetotactic pole-seeking polarity was hypothesized to rely on a yet elusive superimposed mechanism of cellular polarity control, by defining a cellular polarit*y* axis in addition to the magnetosome chain’s magnetic dipole (8, 13). However, the affected motility and loss of swimming polarity are supposedly not directly caused by the absence of *popZ_Mgr_*. In contrast, the aforementioned phenotypes are likely explained by a general impairment of aerotaxis in the Δ*popZ_Mgr_* strain (i.e., due to improper localization of motility-related structures, as discussed below) and as an indirect effect due to formation of short cells that are highly motile, but only weakly aligned within the magnetic field, as well as severe cell elongation, which affects hydrodynamic properties of cells’ propulsion during swimming (as also previously observed for artificially elongated cells caused by cephalexin treatment [6]). Furthermore, we observed an increased tendency of elongated Δ*popZ_Mgr_* cells to form aggregates (Fig. 5E), which might contribute to the strong motility phenotype observed in soft-agar-based assays (Fig. 5B, C, and D). An increased tendency of Δ*popZ* cells to aggregate has also been observed in A. tumefaciens and might be caused by an altered formation of extracellular polysaccharides (25), but in the case of M. gryphiswaldense also due to the helical nature of intertwined elongated cells. It can be further hypothesized that the disturbed aerotactic behavior in the Δ*popZ_Mgr_* strain may be due to a delayed or impaired signal transduction from the chemotactic machinery to the flagellar motors, since some cells contained improperly placed (Fig. 3B) or additional chemosensory clusters (as confirmed by fluorescence microscopy of various methyl-accepting chemotaxis proteins [MCPs] fused to GFP in the Δ*popZ_Mgr_* parent strain [results not shown]) as well as occasional flagella located in nonpolar regions (Fig. 2Aii). Altered localization of MCPs, chemoreceptor-associated histidine kinase CheA, and flagellar basal body proteins FliG and FliM upon *popZ* deletion has been also reported for C. crescentus (17) and A. tumefaciens (25) or in artificially elongated cephalexin-treated E. coli cells (36, 37). However, only a mild effect on motility in swim plate assays has been observed upon *popZ* deletion in A. tumefaciens (25), and artificially elongated E. coli cells were only affected in their swimming speed, but were still able to perform chemotaxis (36). Hence, due to their different flagellation patterns, cell shapes, and chemotactic behaviors, the experimental results among different strains are not directly comparable.

In addition, severe cell elongation upon *popZ* deletion in M. gryphiswaldense resulted in drastically elongated magnetosome chains and a highly increased number of particles per cell (Fig. 2A and B). Our results imply that magnetosome number and chain length are likely directly related to cell length, resembling previously published observations on artificially elongated cephalexin-treated cells (6). Besides, it has recently been shown that increased gene dosage by genomic multiplication of the magnetosome island results in increased particle numbers as well (38), but with several chains running in parallel or cells closely packed with magnetosomes that lack an ordered chain-like arrangement. Presumably, elongated Δ*popZ_Mgr_* cells also possess an increased number of gene copies due to the presence of multiple chromosomes (albeit we were not able to identify distinct individual chromosomes by DAPI staining, without any specific treatment to condense DNA [Fig. 4A]). However, in contrast to the overproducer strain (38), the amount of magnetosomes and gene copies per cell volume in elongated Δ*popZ_Mgr_* cells can be assumed to be roughly in the same range as for the wild type.

Magnetosome synthesis, midcell positioning and proper segregation of magnetosome chains are controlled by the treadmilling behavior of the actin-like MamK, which forms dynamic filaments (6, 7). MamK-dependent repositioning of magnetosome chains was not affected in the Δ*popZ_Mgr_* strain (Fig. 2A and Fig. 6A and B), suggesting that PopZ*_Mgr_* does not play a role in magnetosome organelle segregation or positioning by exerting direct control of the MamK dynamics. Since magnetosome chain segregation is tightly coupled to cell division, it can be hypothesized that PopZ*_Mgr_* may influence magnetosome segregation indirectly—likely by regulating the FtsZ ring localization. Thus, lack of PopZ*_Mgr_* causes unequal cell division and misdistribution of chains during cell division as a side effect.

Most strikingly, a consistent bipolar localization pattern of PopZ in M. gryphiswaldense was observed (Fig. 1, Fig. S2, and Movie S1), contrasting with the reported monopolar-to-bipolar transition in C. crescentus (17, 18) and unipolar localization in A. tumefaciens (23–25). Ortholog substitution experiments between C. crescentus and M. gryphiswaldense (Fig. 7 and Fig. S6) indicated that bipolar PopZ localization is not inherent to the protein itself but rather is host specific. PopZ*_Mgr_* and PopZ*_Cc_* have conserved N and C termini (Fig. S1) and were capable of partially substituting their reciprocal functionalities (Fig. 7). Thus, the observed localization pattern of both orthologs may be explained by a direct interaction with the respective PopZ interactors present in each host, albeit our results also indicate that both orthologs have diverged to an extent that does not allow full conservation of all PopZ-dependent interactions. For C. crescentus, several factors for control of PopZ localization have been discussed (14, 15, 34, 39). Polar localization of PopZ relies on its self-assembly into higher-order structures in DNA-free polar regions, and the unipolar-to-bipolar transition is coupled to the asymmetric distribution of ParA during the cell cycle (14). The chromosome segregation system adaptor protein ParB and the ParA ATPase, which act together to spatially separate replicated chromosomes in C. crescentus (1), might be suitable candidates for control of bipolar PopZ localization in M. gryphiswaldense. Recently, the zinc finger protein ZitP (28, 40) and muramidase homolog SpmX (41) have been described as additional important factors to nucleate new PopZ microdomains in C. crescentus. An ortholog of ZitP (locus tag MGR_3358) is also encoded in the M. gryphiswaldense genome (23% identity and 39% similarity compared to ZitP*_Cc_*, respectively), whereas no protein orthologous to SpmX is present. Further investigation is needed to identify PopZ interactors in M. gryphiswaldense and elucidate how they differ in function from those of other alphaproteobacteria.

In conclusion, protein functions depend on the genetic context, and can be implemented in different ways, even in closely related species. Thus, M. gryphiswaldense also serves as an appropriate and interesting model organism to study the function of cell cycle factors and its coordination with organelle synthesis and segregation. In the near future, these cell-cycle-related studies will also help to understand how polar magnetotaxis is functionally controlled and inherited in MTB.

## MATERIALS AND METHODS

### Bacterial strains, plasmids, and culture conditions.

Bacterial strains and plasmids used in this study are listed in Text S1 in the supplemental material. Cells were grown using previously described standard procedures described in detail in Text S1.

10.1128/mBio.02716-18.10 TEXT S1 Supplemental information containing additional methodological details, strains, plasmids, and primers used in this study. Note strains harboring plasmids or random insertions of P*_tet_*-based expression cassettes are not listed but were generated based on the corresponding parent strains and constructs given in the table. Reverse complementary oligonucleotide sequences are underlined. Restriction sites are indicated in bold. Primers used for blunt end cloning (primers 37, 40, 43, 408, and 411) were phosphorylated with T4 polynucleotide kinase (Thermo Scientific). Download Text S1, DOCX file, 0.1 MB. Copyright © 2019 Pfeiffer et al. 2019 Pfeiffer et al. This content is distributed under the terms of the Creative Commons Attribution 4.0 International license.

### Molecular and genetic techniques.

Oligonucleotides (sequences are listed in Text S1) were purchased from Sigma-Aldrich (Steinheim, Germany). Plasmids were constructed by standard recombinant techniques (as described in Text S1), employing a homologous recombination-based counterselectable system for the construction of in-site deletion and insertion mutants (31) and a Tn*5*-based anhydrotetracycline-inducible expression vector (29, 42) for the construction of transcomplementation and overexpression constructs. All constructs were sequenced by Macrogen Europe (Amsterdam, Netherlands).

### Motility assay.

Motility soft agar assays were performed as described by Popp et al. (13) and in Text S1. Single-cell tracking was performed at 24°C on a Nikon FN1 Eclipse microscope (Fig. S4A) equipped with an S Plan Fluor 20× differential inference contrast (DIC) N1 objective (NA0.5), a dark-field condenser (NA0.95), a pco.edge 4.2 sCMOS camera (PCO), and one pair of coils connected to a Kepco BOP 50-2M power supply. The coil setup was calibrated with a GM08 gaussmeter connected to a TP002HS high-sensitivity transverse probe (Hirst Magnetic Instruments). Hence, 10 µl of cell suspension diluted to an optical density at 565 nm (OD_565_) of 0.01 were pipetted onto a glass slide and covered with a coverslip. Videos were recorded for 40 s at a frame rate of 15 frames per second (fps). Tracking was performed with NIS-Elements 5.1 employing the “spot detection” algorithm and “random motion” model, with the “standard deviation multiplication factor” set to 2.5 and “maximum object speed” set to 80 µm/s. Tracks with 30 or fewer frames and line speeds lower than 2 µm/s were excluded from the analysis. Cells which were tracked due to Brownian motion were deleted manually from the analysis. Values given in Fig. 5A are defined as follows: “line speed” is the length of a straight line from the track origin to the current point (=“line length”) divided by the time elapsed. “Swimming speed” is the track segment length divided by the amount of time elapsed between two positions. “Heading” is the angle between the direction of the velocity vector and the *x* axis. “Alignment” within the magnetic field (along the *x* axis) was calculated in Excel (Microsoft) with the following formula: abs[cos(“heading”)].

### Fluorescence microscopy.

To image fluorescent protein fusions, 3 µl of cell suspension were immobilized on MSR agarose pads (7) and covered with a coverslip. For fluorescent staining, 20 µl of cell suspension was mixed with either 10 µl of a Nile red solution (0.5 µg/ml in dimethyl sulfoxide [DMSO]) or 6 µl of MM4-64 (an FM4-64 derivative [16 µM in DMSO]) and/or 10 µl of a DAPI solution (50 µg/ml). Conventional epifluorescence microscopy was performed on an Olympus BX81 microscope equipped with a 100× UPLSAPO100×O objective (NA1.4) and an Orca-ER camera (Hamamatsu). Time-lapse imaging and fluorescence recovery after photobleaching (FRAP) were performed on a Deltavision Elite system (GE Healthcare) equipped with a U-Plan S-Apo 100× oil PSF objective (NA1.4) and a CoolSnap HQ2 charge-coupled device (CCD) camera as described previously (7). Additional time-lapse series were acquired on a Nikon Eclipse Ti2-E microscope equipped with a CFI SR Apo TIRF AC 100×H oil objective (NA1.49) and Retiga R1 CCD camera (QImaging). Further methodological details with respect to image deconvolution and the different systems used for epifluorescence microscopy, FRAP, and time-lapse imaging are given in Text S1.

### Structured illumination microscopy.

3D-SIM (striped illumination at 3 angles and 5 phases) was performed on a Nikon Eclipse Ti2-E N-SIM E fluorescence microscope equipped with a CFI SR Apo TIRF AC 100×H NA1.49 oil lens objective. Samples (3 µl of FM4-64-stained cell suspension) were immobilized on MSR agarose pads (7). High-precision coverslips of 0.17 mm thickness (no. 15H; Marienfeld) and immersion oil (type F 30cc; Nikon) with a refractive index of 1.518 (at 23°C) were used to minimize sample-induced spherical aberration. Samples were sealed with wax to provide long-term stability and avoid sample drift during imaging. Calibration of the SIM grating focus and motorized temperature change objective correction collar was performed at 22°C with a bead sample (T-7279 TetraSpeck microspheres) for high-quality image reconstructions at around 115-nm lateral (xy) spatial resolution with 32.5-nm reconstructed image pixel spacing. System and samples were preequilibrated at 22°C before imaging to avoid temperature-induced changes in the refractive index. Fast piezo stage z-series images were taken at a total thickness of 1.5 to 2.0 µm with 200-nm z-step spacing with raw frame exposure times in the range 100 to 150 ms, avoiding detector saturation of the 16-bit 1.5-electron read noise Orca Flash4.0 LT Plus sCMOS camera (Hamamatsu). EM700/75 and EM525/50 emission filters and fluorescence excitation with 561- and 488-nm laser lines at 80% laser power were used for imaging of FM4-64 and PopZ*_Mgr_*-GFP, respectively. 3D-SIM image reconstruction was performed in NIS-Elements 5.01 (Nikon) using the “stack reconstruction” algorithm with the following parameter settings. The “illumination modulation contrast” was set either to “auto” for the FM4-64 channel or to custom values between 1.5 and 3.3 for the GFP channel. The “high resolution noise suppression” was set to 0.1.

### Image analysis.

Images were processed and analyzed with ImageJ Fiji v1.50c (43). Demographs and line plots (Fig. 1 and Fig. S6) were constructed by manually measuring the fluorescence intensity profiles in Fiji and processing the data in R (version 3.3.1 [http://www.r-project.org]), with the cell profiles script (21 [http://github.com/ta-cameron/cell-profiles]) and ggplot2 package (version 2.1.0; Hadley Wickham, Department of Statistics, Rice University [https://ggplot2.tidyverse.org/]). Intensity profiles either were oriented with the fluorescent cell pole toward the left (E. coli and R. sphaeroides), or random sorting was applied (M. gryphiswaldense and R. rubrum). In the case of C. crescentus, the stalk was used as the old pole reference. To confidently and accurately identify stalks, FM4-64 staining (which in our hands did not always stain all cells) and DAPI staining (which we found to unspecifically stain stalks) were used, in addition to DIC microscopy.

The corrected mean cell fluorescence (Fig. 4) was determined in ImageJ Fiji by estimation of the “mean gray values” (=the sum of the gray values of all the pixels in the selection divided by the number of pixels) in selected cell outlines subtracted by the “mean gray value” of the background. For the selection and quantification of cell poles, circular outlines with a diameter of 450 nm were used. The background was estimated within square outlines with a size of 20 by 20 pixels.

### Transmission electron microscopy and cryo-electron tomography.

Conventional transmission electron microscopy (TEM) and cryo-electron tomography (CET) were performed in a similar manner as described previously (7). Detailed instructions are given in Text S1.

### Statistical analysis.

Statistical analysis was performed in Prism 7.04 (GraphPad) as described in the respective legend to each figure. Data sets were tested for normality using the D’Agostino and Pearson, Shapiro-Wilk, and Kolmogorow-Smirnov tests.
