# Supplementary material for: The Polar Organizing Protein PopZ Is Fundamental for Proper Cell Division and Segregation of Cellular Content in Magnetospirillum gryphiswaldense
Source: mBio. 2019 Mar 12;10(2):e02716-18. doi: 10.1128/mBio.02716-18 (PMC6414705; doi:10.1128/mBio.02716-18)
Supplement: FIG S1 [file mBio.02716-18-sf001.pdf]

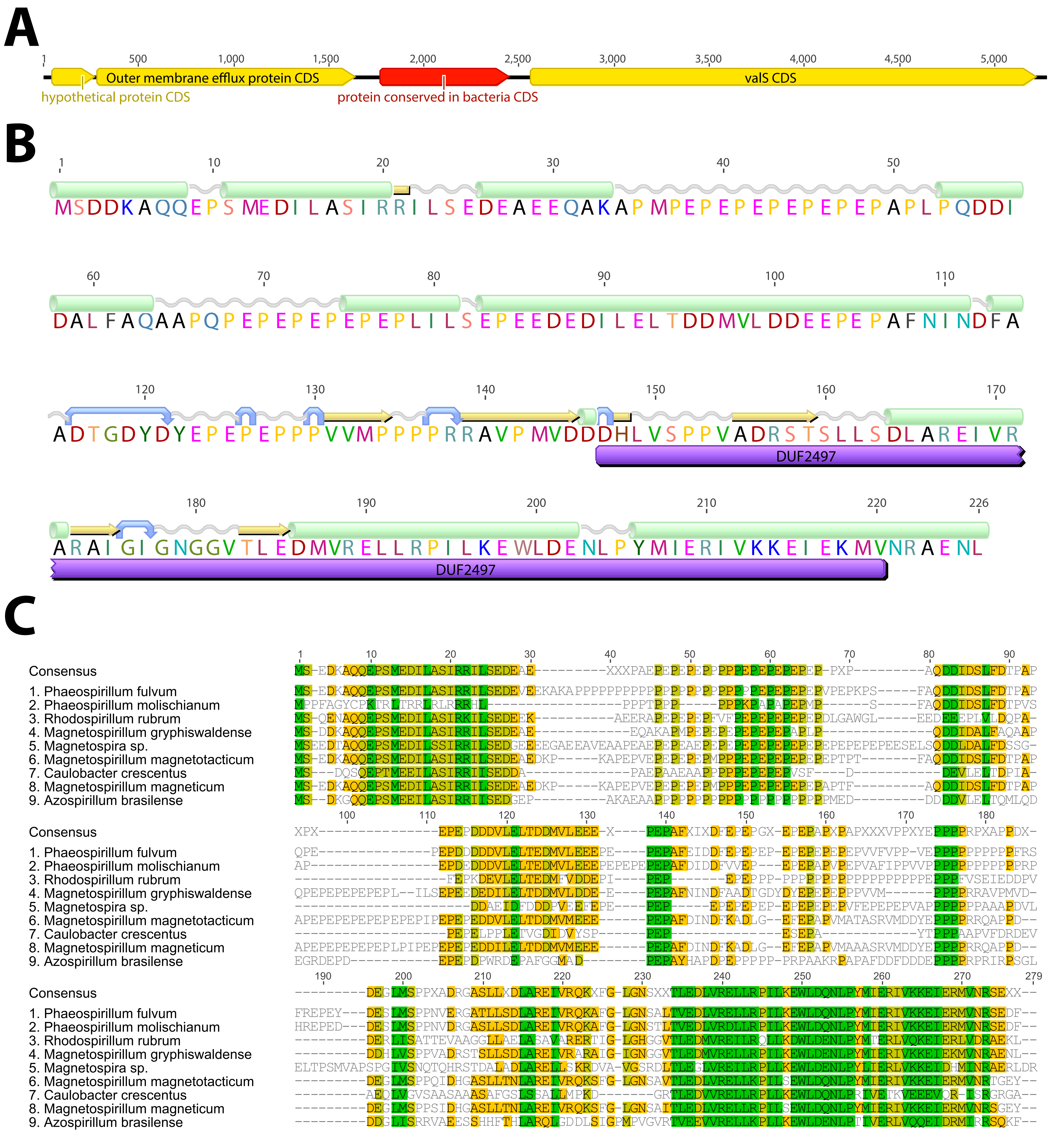

D

Identity / Similarity	*Phaeospirillum fulvum*	*Phaeospirillum molischianum*	*Rhodospirillum rubrum*	*Magnetospirillum gryphiswaldense*	*Magnetospira sp.*	*Magnetospirillum magnetotacticum*	*Caulobacter crescentus*	*Magnetospirillum magneticum*	*Azospirillum brasilense*
Phaeospirillum fulvum	100%	73.50%	47.63%	61.31%	51.62%	67.61%	36.79%	67.48%	42.52%
Phaeospirillum molischianum	73.11%	100%	36.81%	51%	39.91%	56.77%	30.15%	57.01%	31.62%
Rhodospirillum rubrum	57.70%	49.46%	100%	48.98%	44.82%	45.78%	38.38%	45.55%	39.82%
Magnetospirillum gryphiswaldense	65.23%	56.98%	59.49%	100%	48.40%	64.68%	37.22%	65.81%	41.85%
Magnetospira sp.	59.13%	46.59%	56.63%	50.89%	100%	45.80%	36.01%	45.58%	38.47%
Magnetospirillum magnetotacticum	72.04%	59.85%	54.83%	71.32%	50.17%	100%	40.85%	95.06%	41.10%
Caulobacter crescentus	45.87%	43.72%	54.48%	51.25%	44.44%	47.67%	100%	41.52%	40.49%
Magnetospirillum magneticum	70.96%	59.49%	54.83%	72.04%	49.82%	96.77%	48.38%	100%	41.27%
Azospirillum brasilense	51.61%	43.72%	54.12%	54.48%	45.16%	53.04%	50.89%	53.76%	100%
