# Supplementary figures and images for: The Polar Organizing Protein PopZ Is Fundamental for Proper Cell Division and Segregation of Cellular Content in Magnetospirillum gryphiswaldense
Source: mBio. 2019 Mar 12;10(2):e02716-18. doi: 10.1128/mBio.02716-18 (PMC6414705; doi:10.1128/mBio.02716-18)

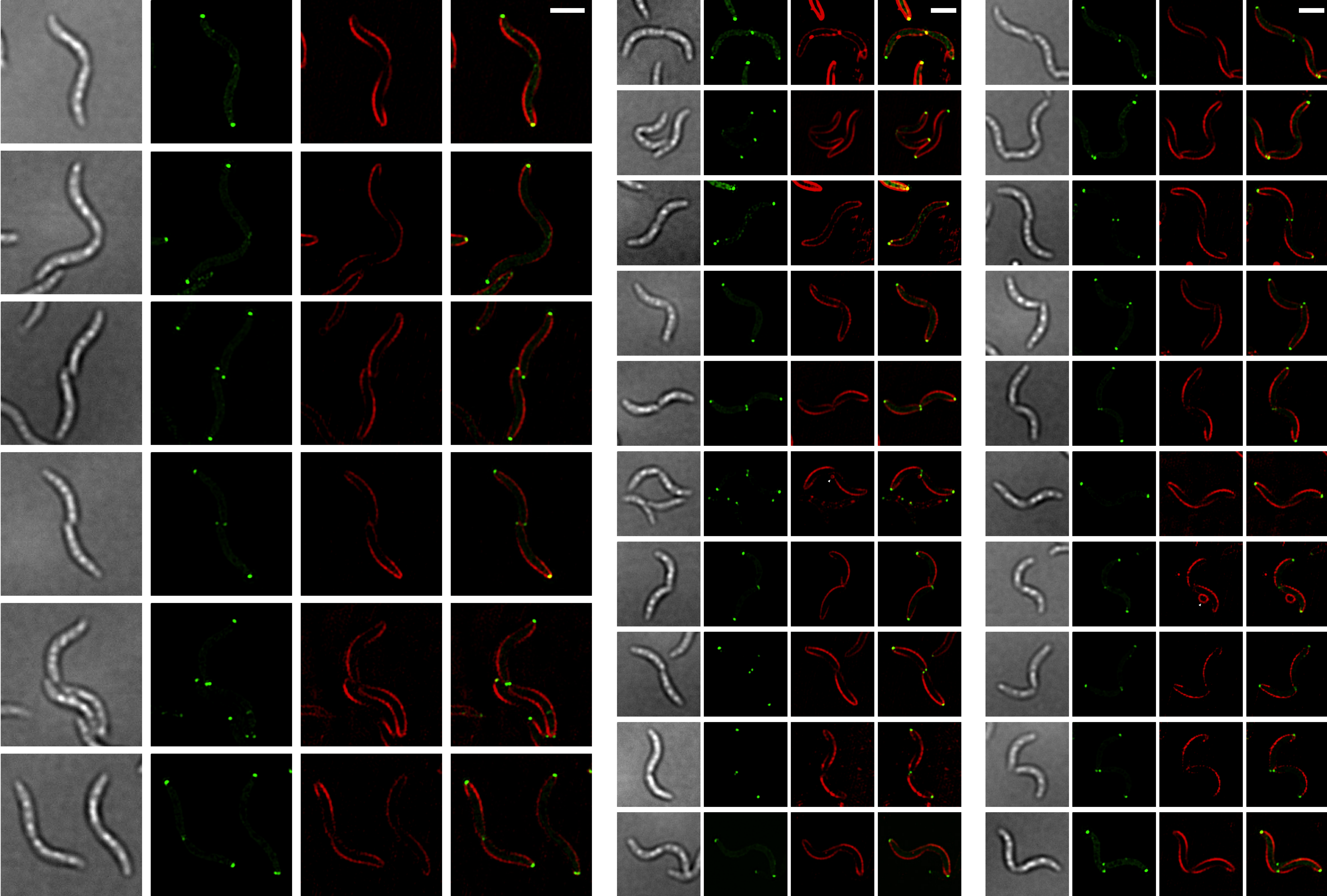

Supplement: FIG S2 [file mBio.02716-18-sf002.pdf]

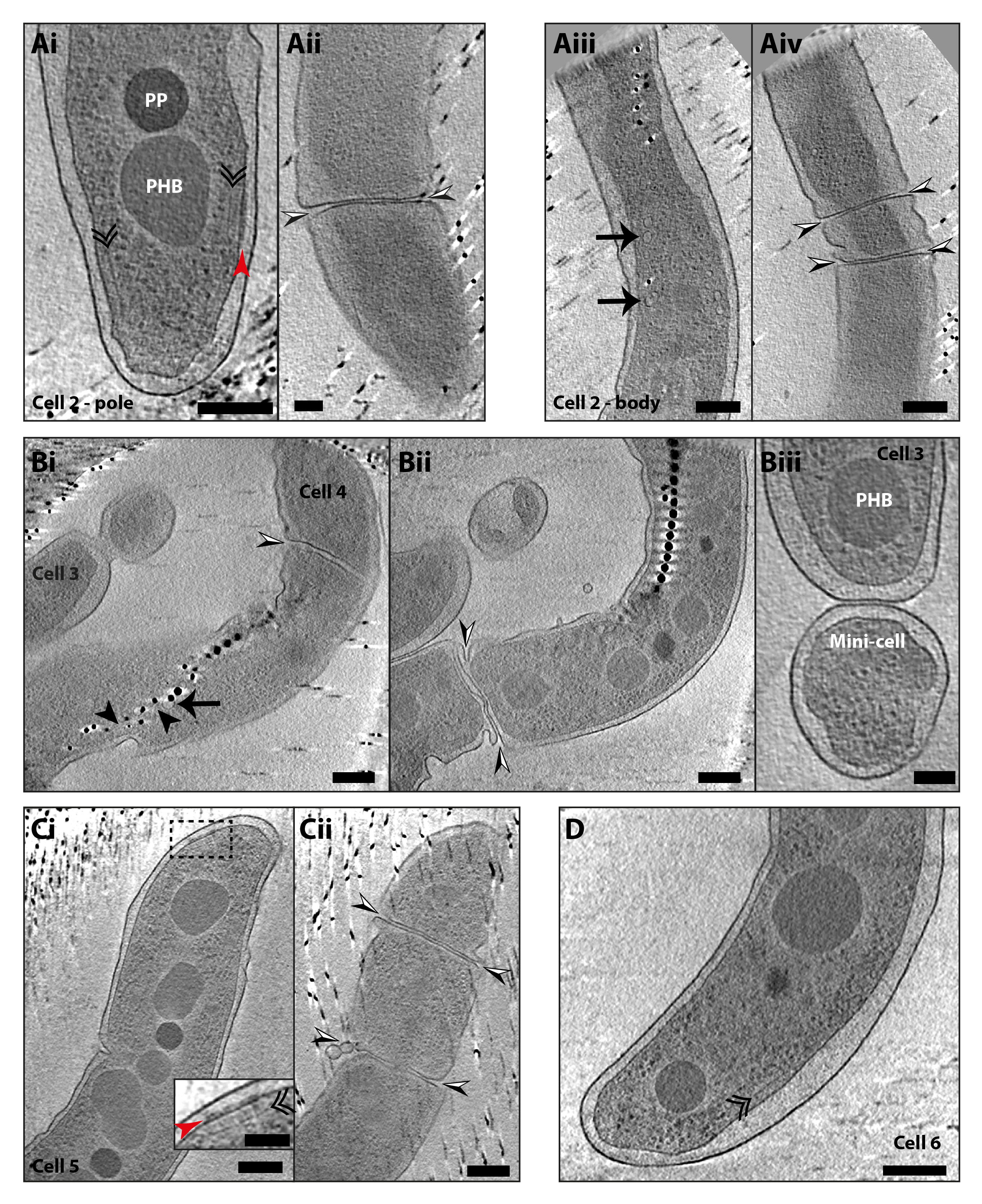

Supplement: FIG S3 [file mBio.02716-18-sf003.jpg]

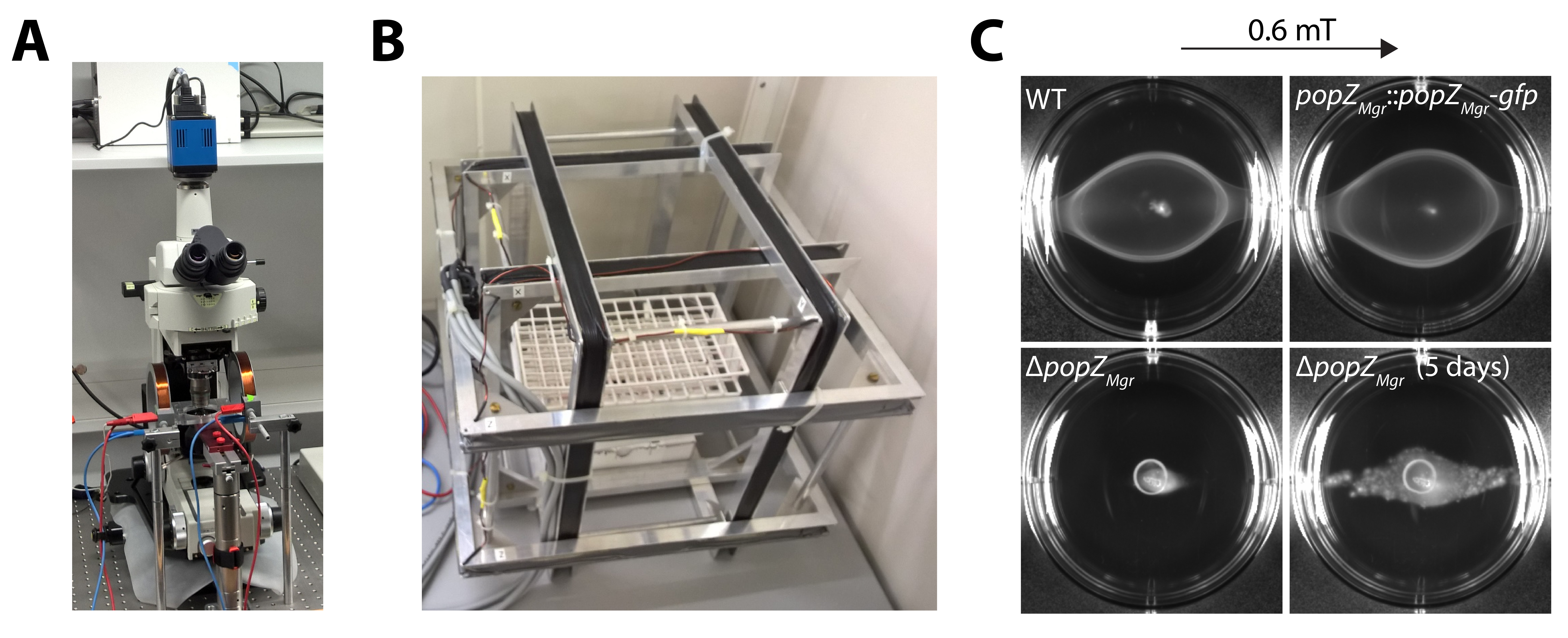

Supplement: FIG S4 [file mBio.02716-18-sf004.jpg]

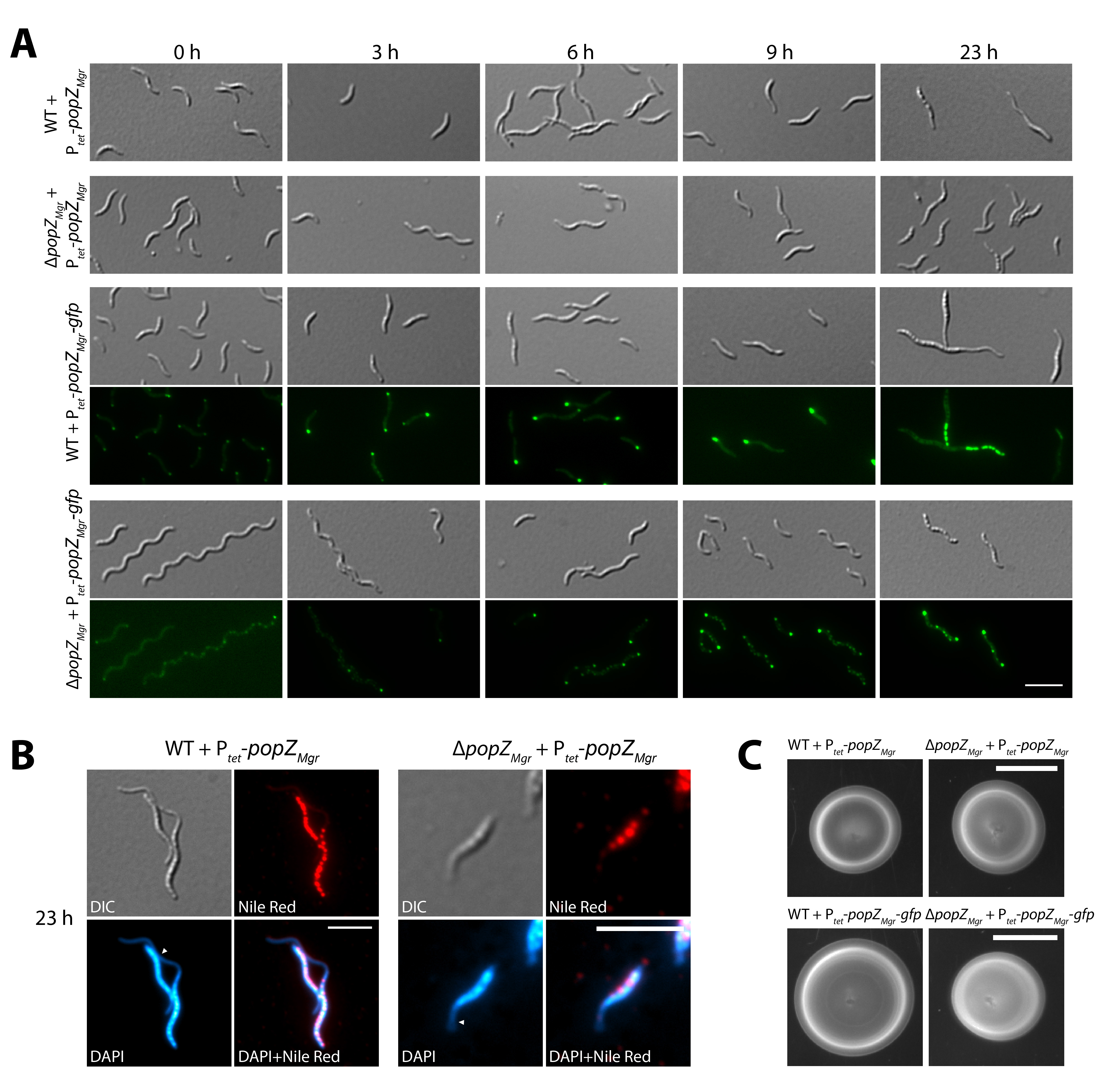

Supplement: FIG S5 [file mBio.02716-18-sf005.jpg]

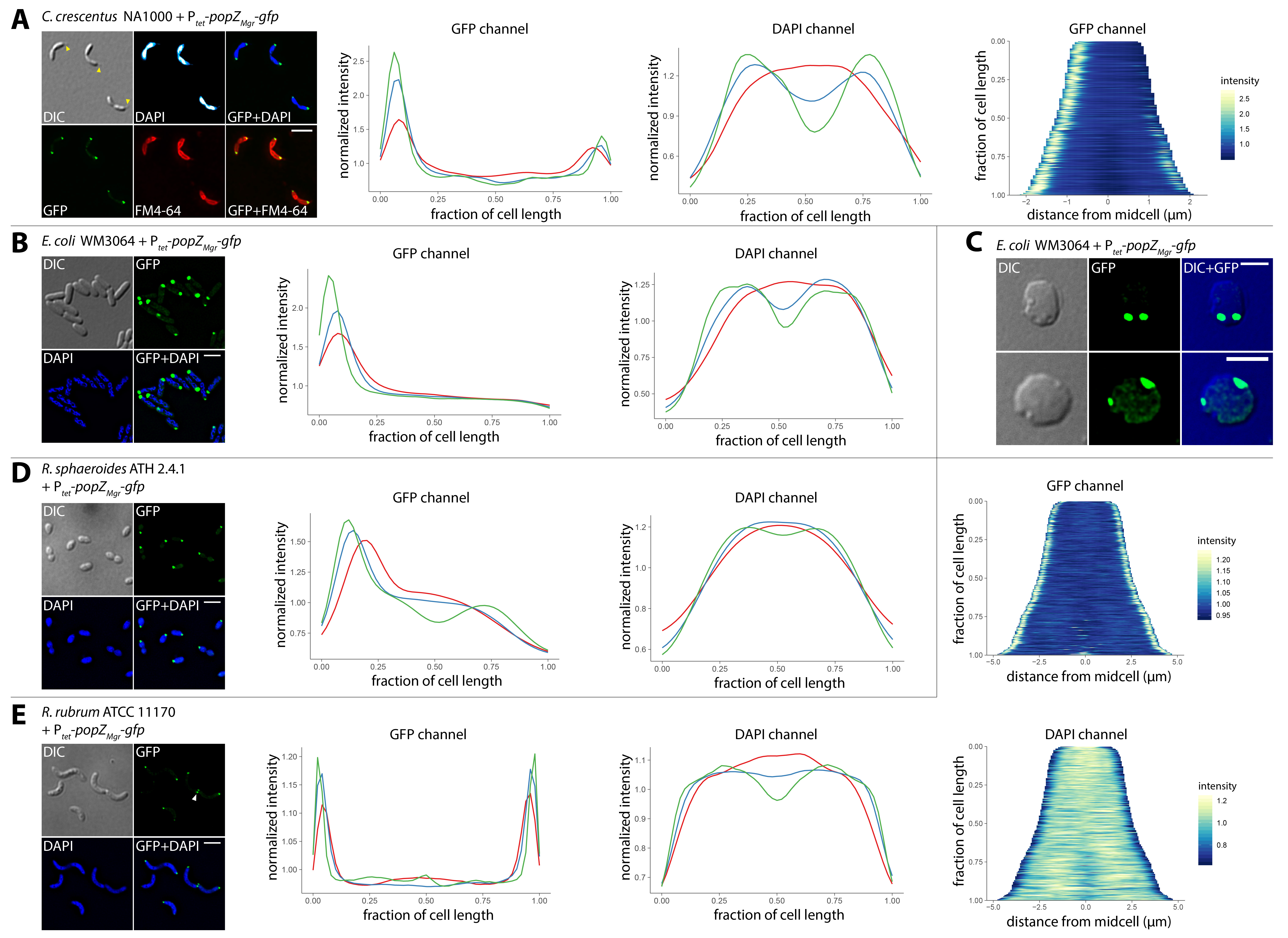

Supplement: FIG S6 [file mBio.02716-18-sf006.jpg]
