# Supplementary material for: The Polar Organizing Protein PopZ Is Fundamental for Proper Cell Division and Segregation of Cellular Content in Magnetospirillum gryphiswaldense
Source: mBio. 2019 Mar 12;10(2):e02716-18. doi: 10.1128/mBio.02716-18 (PMC6414705; doi:10.1128/mBio.02716-18)
Supplement: TEXT S1 [file mBio.02716-18-s0001.docx]

Supplemental Text

# Supplemental Materials and Methods

## Bacterial strains and culture conditions

*E. coli* strains were cultivated in lysogeny broth (LB) medium at 37°C and shaking (180 rpm). For cultivation of *E. coli* WM3064 (W. Metcalf, unpublished) the medium was supplemented with 1 mM DL-α,Ɛ-diaminopimelic acid (DAP). *C. crescentus* strains were grown at 28°C in PYE medium [(1)](#_CTVL00185695cb420914a0b991221bd1036f51f) or HIGG minimal medium with limiting amounts of phosphate [(2)](#_CTVL001ecc70a5593eb4a01bfee3ac3875a6460) on a shaker (120 rpm). *R. sphaeroides* and *R. rubrum* strains were grown in YPS medium [(3)](#_CTVL001c219f0f79ac54a6e9d1d2df7faa833f7), either photoheterotrophically in closed screw cap tubes with illumination from a light bulb or chemoheterotrophically in shake flasks at 28°C and 120 rpm. *M. gryphiswaldense* strains were grown microaerobically (2% headspace oxygen) in modified flask standard medium (FSM, [(4)](#_CTVL00102a8fe5a643f46e98a0a892bc35dd3c9)) at 28°C in 6-well plates without agitation or in hungate tubes with moderate shaking (120 rpm). Optical density and magnetic response (Cmag) of *M. gryphiswaldense* cultures were estimated photometrically at 565 nm as reported previously [(5)](#_CTVL00121965ca565f0446eabd14ec41007a3e1). For routine comparison of growth, *M. gryphiswaldense* strains were grown in 24-well plates (Sarstedt, Nümbrecht) in each 1 ml of culture volume in a microplate reader (infinite 200Pro, Tecan, Switzerland) with automated reading of absorbance (560 nm) and when appropriate GFP fluorescence (excitation: 485 nm, emission: 535 nm) under aerobic conditions at 28°C with shaking (142 rpm). Values were corrected using FSM medium as blank. To ensure reproducibility, precultures were grown for at least 3 passages before start of the experiment and diluted to the same optical density. Media were solidified by the addition of 1.5% (wt/vol) agar. When appropriate, selection was achieved by addition of kanamycin at a final concentration of 5 µg/ml (*M. gryphiswaldense*), 25 µg/ml (*E. coli* and *C. crescentus*)*,* 10 µg/ml (*R. sphaeroides*) or 20 µg/ml (*R. rubrum*), respectively.

For fluorescence microscopy, *M. gryphiswaldense* strains were either grown under undefined microoxic conditions in 15 ml polypropylene tubes with sealed screw caps in a culture volume of 10 ml or in 6-well plates in a culture volume of 3.5 ml under defined microoxic conditions (2% O_2_). *E. coli*, *C. crescentus*, *R. sphaeroides* and *R. rubrum* strains were grown under aerobic conditions in a volume of 10 ml in shake flasks using the same media, temperature and shaker settings as listed above.

## Construction of in-site deletion and insertion mutants

Among the representative magnetotactic members, PopZ orthologs were found in vibroid strain *Magnetovibrio blakemorei* MV-1, in *Terasakiella sp*. PR1 and in all magnetospirilla, whereas PopZ is absent in the genus *Magnetococcus*. A markerless in-frame deletion mutant of the respective putative *popZ_Mgr_* gene in *M. gryphiswaldense* (GenBank accession number: CU459003, locus_tag: mgr_3089) was constructed using a homologous recombination based counterselectable system described previously [(6)](#_CTVL001e43ef733da3b4f3ebc4196a21636a63e). Since the predicted protein lacked the first 11 amino acids in comparison with PopZ*_Cc_* (*C. crescentus* CB15 genome, locus_tag: CC_1319) and with the derived peptide of the open reading frame of the *popZ_Mgr_* gene in another version of the *M. gryphiswaldense* genome (GenBank accession number: GCA_000513295.1, locus_tag: mgmsrv2_1075), a start-codon 33 nt upstream was considered to be more likely. For construction of the deletion plasmid homologous regions of ~700 bp located directly up- and downstream of the *popZ_Mgr_* locus were amplified with Phusion DNA polymerase (Thermo Scientific) with primer pairs 43/44 and 40/45. Both fragments were fused via overlap extension PCR. The reverse primer for the upstream fragment and the forward primer of the downstream fragment included the first three and last three codons of the *popZ_Mgr_* gene as overlapping sequence, which results in the expression of a ‘nonsense‘ peptide after deletion of the gene. The deletion fragment was ligated ‘blunt end’ into pORFM cut with EcoRV and dephosphorylated with FastAP thermosensitive alkaline phosphatase (Thermo Scientific). After conjugative transfer of the resulting deletion plasmid pORFM-Δ*popZ_Mgr_* to *M. gryphiswaldense* and *galK*-based counterselection, colonies were screened for deletion of *popZ_Mgr_* with primers 42/46. Deletion of *popZ_Mgr_* was confirmed by sequencing of the respective PCR product.

Construction of a plasmid for insertion of *gfp* a the native chromosomal *popZ_Mgr_* locus (pORFM-*popZ_Mgr_-gfp*) was conducted in the same manner. Therefore, fragments up- and downstream of the *popZ_Mgr_* stop codon were amplified with primer pairs 37/38 and 39/40 and fused via overlap extension PCR, resulting in a fusion with *gfp* between both fragments. A codon-optimized version of *gfp* (*magegfp*) [(7)](#_CTVL001ec18adb5cb8143818633f24319064057) with a sequence coding for a 4-helix linker (ASLAEAAAKEAAAKEAAAKEAAAKAAAVH) was amplified with primers 5/6. Insertion of *gfp* at the *popZ_Mgr_* locus after *galK*-based counterselection was confirmed by PCR with primers 41/42 and by sequencing of the respective PCR product. Of note, expression of the *popZ_Mgr_-gfp* fusion from the native chromosomal popZ*_Mgr_* locus (as sole source of PopZ*_Mgr_*) did not affect cell morphology, growth or motility (Fig. 1, Fig. 2D, E and Fig. 5B, D).

To replace *popZ_Mgr_* chromosomally with a fragment coding for a functional mCherry-PopZ*_Cc_* fusion [(8)](#_CTVL001da530333c0744889962fe4fc0f108a39), the respective gene fragment was amplified from a *C. crescentus* strain harboring a *mcherry-popZ_Cc_* fusion (Table S1) with primers 414/415. The resulting PCR product was sandwiched between two PCR fragments (amplified with primer pairs 43/416 and 40/417, respectively) located up- and downstream of *popZ_Mgr_* via overlap-extension PCR. Subsequently, the resulting fragment was cloned blunt-end into EcoRV-digested and dephosphorylated pORFM vector and the final plasmid pORFM-*mcherry*-*popZ_Cc_* was transferred to the *M. gryphiswaldense* wildtype strain via conjugation. Chromosomal replacement of *popZ_Mgr_* with *mcherry*-*popZ_Cc_* after counterselection was confirmed by PCR with primers 42/46 and by sequencing of the respective PCR product.

The reciprocal experiment (replacement of *popZ_Cc_* in *C. crescentus* with *popZ_Mgr_*-*gfp*) was conducted by amplification of *popZ_Mgr_*-*gfp* with primers 6/37 and subsequent fusion between two fragments located up- and downstream of the *popZ_Cc_* locus (amplified with primers 408/409 and 410/411, respectively). The resulting fragment was cloned into pNTPS138 (M.R.K. Alley, unpublished) cut with EcoRV. Finally, the resulting plasmid pNTPS138-*popZ_Mgr_*-*gfp* was transferred to the *C. crescentus* NA1000 wildtype strain via conjugation. Counter-selection was achieved by addition of 3% sucrose. Replacement of *popZ_Cc_* with *popZ_Mgr_*-*gfp* was confirmed by PCR with primers 412/413 and by DNA sequencing.

## Transcomplementation of Δ*popZ_Mgr_* and overexpression of *popZ_Mgr_*

For construction of plasmids for transcomplementation of the Δ*popZ_Mgr_* strain and overexpression of *popZ_Mgr_* (or *popZ_Mgr_-gfp*) in *M. gryphiswaldense*, *popZ_Mgr_* and *popZ_Mgr_-gfp* were amplified from strains *M. gryphiswaldense* wildtype and *popZ_Mgr_*::*popZ_Mgr_-gfp* with primer pairs 102/103 and 83/102, respectively. Both fragments were ligated into pJH40 (a pBAM-based anhydrotetracycline inducible expression vector [(9](#_CTVL0018cbff3591f9b4934b41427d8ecb6f0ed)[, 7](#_CTVL001ec18adb5cb8143818633f24319064057)[, 10)](#_CTVL001fac8970ab7af40628948f7a8ea7b1ff1)) after digestion with NdeI and BamHI. The resulting plasmids pBAM-P*_tet_*-*popZ_Mgr_* and pBAM-P*_tet_*-*popZ_Mgr_-gfp* were transferred to different strains via conjugation using *E. coli* WM3064 as donor. After Tn5-based random single copy chromosomal insertion of the expression cassette, gene expression was induced by transfer of cells to fresh medium and addition of 50 ng/ml anhydrotetracycline.

## Motility assay

For the preparation of FSM [(4)](#_CTVL00102a8fe5a643f46e98a0a892bc35dd3c9) swim agar plates, 0.2% (wt/vol) agar was used and the concentration of lactate (carbon source) was lowered tenfold to 1.2 mM. Five microliters of culture was pipetted into the agar, and plates were incubated at 28°C for 2-5 days. 120 ml of sterile soft agar was poured into large petri dishes (14 cm diameter) and 5 ml into each well of a 6-well plate (3.5 cm diameter). Plates were documented with a ChemiDoc XRS+ imager (Bio-Rad) and Image Lab 5.2.1 software at 1.5 s exposure time without an additional filter. For gradient tubes 10 ml of 0.3% sterile motility agar (FSM with tenfold reduced potassium lactate concentration) was poured into glass tubes and mixed with 100 µl of a microoxically grown overnight culture. Cultures were adjusted to the same density prior to inoculation. For enrichment of cells with NS or SS swimming polarity bias, cells were passaged in Hungate tubes, which were incubated within a 0.6 mT magnetic field applied in *z*-direction (Fig. S4B), as described in Popp *et al.* [(11)](#_CTVL001ff61da501e84466191e73a4a3777a21d).

## Epifluorescence microscopy

Samples were imaged with an Olympus BX81 microscope equipped with a 100x UPLSAPO100XO objective (NA1.40) and an Orca-ER camera (Hamamatsu). Exposure times were set between 50-800 ms depending on the fluorophore using appropriate filter sets. Images were captured using Olympus Xcellence 1.1 software. 3D Deconvolution of *z*-stacks acquired on the Olympus microscope was performed with DeconvolutionLab 2.0.0 and PSF generator 1.1.1.2 (Biomedical Imaging Group, EPFL) [(12)](#_CTVL001dd20f3d2337d42e2839cc6629a07e3d6), employing 200 iterations of the Richardson-Lucy algorithm [(13](#_CTVL0015b3a51d779bb4d8996e7b8ce4b0f43e4)[, 14)](#_CTVL001898644d5553b4af9bbc860d615693f51) and the following settings for PSF generation: Born & Wolf 3D optical method; computation accuracy, best; refractive index immersion oil, 1.518 (Olympus Immersion oil Type-F); emission wavelength: 440 nm (DAPI filter cube), 535 nm (GFP filter cube), 640 nm (FM4-64 filter cube); pixel size, 64 nm; *z*-step width, 150 nm). 2D deconvolution of single plane images acquired on the Olympus microscope was performed with NIS Offline deconvolution 4.51 (Nikon), with similar settings (noise level: clear).

## Time-lapse microscopy

Time-lapse series of the *M. gryphiswaldense* wildtype, Δ*popZ_Mgr_* and *popZ_Mgr_*::*popZ_Mgr_*-*gfp* strains (Fig. 1 and Fig. 2) were acquired on a Deltavision Elite System (GE Healthcare) using an Olympus IX71 microscope equipped with InsightSSI Illumination System and a CoolSnap HQ2 CCD camera. Time-lapse imaging was performed using a ‘MSR agarose pad’ (as described in Toro-Nahuelpan *et al.* [(15)](#_CTVL001bc4c707275db4f0ea61901c8d298d69a)) at 30°C with a hardware based ‘Ultimate-Focus’ autofocus. Images were collected with a 100x Oil PSF objective (U-PLAN S-APO 100x Oil, 1.4NA, 0.12 WD) using the FITC filter set for imaging of PopZ*_Mgr_*-GFP and SoftWoRx Suite 2.0 software. Time-lapse series of the strains *M. gryphiswaldense popZ_Mgr_*::*mcherry*-*popZ_Cc_* and *C. crescentus* *popZ_Cc_*::*popZ_Mgr_*-*gfp* (Fig. 7) were acquired at 28°C on a Nikon Eclipse Ti2-E microscope equipped with a CFI SR Apo TIRF AC 100xH NA1.49 Oil objective, external phase contrast and perfect focus system (Nikon), incubation chamber (Tokai Hit), Spectra X light source (Lumencor) and a Retiga R1 CCD camera (QImaging). In case of *C. crescentus*, ‘PYE agarose pads’ (1% (w/v) agarose in PYE medium) were used. DAPI/FITC/Cy3/Cy5 Quad and CFP/YFP/mCherry Triple filter sets were used for imaging of PopZ*_Mgr_*-GFP and mCherry-PopZ*_Cc_*, respectively. Images were collected using NIS-Elements 5.01.

## Photokinetic analysis

Fluorescence recovery after photobleaching (FRAP) was performed as per Toro-Nahuelpan *et al.* [(15)](#_CTVL001bc4c707275db4f0ea61901c8d298d69a). Cells were mounted on ‘MSR agarose pads’ and imaged with a Delta Vision Elite system (GE Healthcare, see above). For mCherry-MamK imaging the mCherry filter set was used at 32% SSI and 150 ms exposure. Bleaching: 561 nm laser line (50 mW) at 10% power, 70% of laser in TIRF mode (only to decrease laser power, TIRF imaging was not performed) and a single pulse for 4 ms. Cells were imaged every 30 s. The laser event was always placed after the first image. Half-time fluorescence recoveries (t _½_) were calculated independently per each bleached cell and averaged in order to obtain the SEM for the cells community. Additionally, each FRAP related plot shows the SD per each time point. FRAP data analysis was performed as described in Toro-Nahuelpan *et al.* [(15)](#_CTVL001bc4c707275db4f0ea61901c8d298d69a). Briefly, images were aligned and the integrated density over time determined from the regions of interest using Fiji software. The average values of 23 cells of several biological replicates were plotted. Recovery rates were determined by fitting the data obtained for the bleached region to the single exponential function: F(*t*) = A [1- exp(-*k* * *t*)] + F(0). Where F(*t*) is the fluorescence at time *t*, A the maximum intensity, *k* the rate constant and F(0) the relative fluorescence intensity at *t* = 0 min.

## Transmission electron microscopy

For transmission electron microscopy (TEM) analysis, cells were grown at 28°C under microaerobic conditions (2% O_2_). If necessary cells were fixed by addition of formaldehyde (1.5%) and incubation for at least one hour at 4°C. Cells were concentrated by centrifugation of 1 ml of culture (OD_565_ ~0.15) at 3.500 *g* for 5 min and followed by resuspension in ~50 µl of residual medium. Afterwards cells were adsorbed onto carbon coated copper mesh grids (CF200-CU, Electron Microscopy Sciences, Pennsylvania) and washed two times with millipore water. Images were recorded with a EM Zeiss 902A microscope at 80 kV accelerating voltage.

## Plunge-freezing vitrification

5 μl of *M. gryphiswaldense* culture were mixed with 2 μl of BSA-coated 15 nm colloidal gold particles (Sigma, used for subsequent alignment purposes) and added on glow-discharged Quantifoil holey carbon copper grids (Quantifoil Micro Tools GmbH, Jena). The mixture was blotted and embedded in vitreous ice by plunge freezing into liquid ethane (< −170°C). The grids were stored in sealed boxes in liquid nitrogen until used.

## Cryo-electron tomography

Tomography was performed under low-dose conditions using a Tecnai G^2^ Polara transmission electron microscope (FEI) equipped with a 300 kV field emission gun, and a Gatan GIF 2002 post-column energy filter. A 3838 x 3710 Gatan K2 Summit Direct Detection Camera operated in counting and dose-fractionation mode was used for imaging. Data collection was performed at 300 kV, with the energy filter operated in the zero-loss mode (slit width of 20 eV). Tilt series were acquired using Serial EM software [(16)](#_CTVL001670ff513d154450182611016916562c7). The specimen was tilted about one axis with 1.5° increments over a typical total angular range of ± 60°. The cumulative electron dose during the tilt series was kept below 150 e^-^ Å^-2^. To account for the increased specimen thickness at high tilt angles, the exposure time was multiplied by a factor of 1/cos α. Pixel size at the specimen level was 5.22 Å at an EFTEM magnification of 22500x. Images were recorded at nominal −5 μm defocus.

## Tomogram reconstruction and segmentation

Tomograms were reconstructed in the IMOD package [(17)](#_CTVL001e12fd78903194651928644cf4ffdb3a1). Tomographic reconstructions from tilt series were performed with the weighted back-projection with IMOD software using gold particles as a fiducial marker. Aligned images were binned to the final pixel size of 31.32 Å. For tomographic reconstruction, the radial filter options were cut off: 0.5 and fall off: 0.05. The dataset for this study consisted of 7 tomograms from 6 cells. Tomograms were treated with an anisotropic nonlinear diffusion denoising algorithms to improve signal-to-noise ratio. Segmentation of the tomogram was done with Amira software on binned volumes with a voxel size of 31.32 Å. Membrane segmentation was done using the software TomoSegMemTV and a complementary package, SynapSegTools, both for Matlab [(18)](#_CTVL001bdf1bddc72c144a2bf0b864783a4ee5f). Tomograms slices were obtained using 3dmod software from the IMOD package.

Table S1 Strains, plasmids and primers used in this study. Note, strains harboring plasmids or random insertions of P*_tet_*-based expression cassettes are not listed but were generated based on the according parent strains and constructs given in the table. Reverse complementary oligonucleotide sequences are underlined. Restriction sites are indicated in bold. Primers used for ‘blunt end’ cloning (Primer No. 37, 40, 43, 408, 411) were phosphorylated with T4 polynucleotide kinase (Thermo Scientific).

| Strain or vector | | | | Relevant characteristic(s) | | **Reference and/or source** |
| --- | --- | --- | --- | --- | --- | --- |
| *Strains* | | | |  | |  |
|  | *E. coli* | | |  | |  |
|  |  | DH5α | | Host for cloning; F^–^ *endA1* *glnV44* *thi-1* *recA1* *relA1* *gyrA96* *deoR* *nupG* *purB20* φ80d*lacZ*ΔM15 Δ(*lacZYA-argF*)U169, hsdR17(*r_K_*^–^*m_K_*^+^), λ^–^ | | [(19)](#_CTVL001d9749bf3f33b443bb68c3a66c4b0b4ef) |
|  |  | WM3064 | | Conjugation strain; *thrB1004 pro thi rpsL hsdS lacZ*ΔM15 RP4-1360 Δ(*araBAD*)567 Δ*dapA*1341::[*erm pir*] | | William Metcalf at UIUC, unpublished |
|  | | | | | | |
|  | *M. gryphiswaldense* | | |  | |  |
|  |  | MSR-1 R/S | | wildtype | | [(20)](#_CTVL0017b5a45872fde4c1aa13780dde6dc65ad) |
|  |  | Δ*popZ_Mgr_* | | *popZ_Mgr_* deletion strain | | This study |
|  |  | *popZ_Mgr_*::*popZ_Mgr_-gfp* | | strain with insertion of *gfp* at the chromosomal *popZ_Mgr_* locus | | This study |
|  |  | *popZ_Mgr_*::*mcherry*-*popZ_Cc_* | | chromosomal site-specific replacement of *popZ_Mgr_* with *mcherry*-*popZ_Cc_* | | This study |
|  | | | | | | |
|  | *C. crescentus* | | |  | |  |
|  |  | NA1000 | | wildtype | | [(21)](#_CTVL0019f7eaf6aa09746fc955f1adaa10a34fe) |
|  |  | Δ*popZ_Cc_* | | *popZ_Cc_* deletion strain | | [(22)](#_CTVL0019be84854686b4dd5a0c2622be79e877a) |
|  |  | *popZ_Cc_*::*mcherry*-*popZ_Cc_*; *parB*::*cfp*-*parB* | | source of *mcherry*-*popZ_Cc_* | | [(8)](#_CTVL001da530333c0744889962fe4fc0f108a39) |
|  |  | *popZ_Cc_*::*popZ_Mgr_*-*gfp* | | chromosomal site-specific replacement of *popZ_Cc_* with *popZ_Mgr_*-*gfp* | | This study |
|  | | | | | | |
|  | *R. rubrum* ATCC 11170 | | | wildtype | | DSM467 |
|  | *R. sphaeroides* ATH 2.4.1 | | | wildtype | | DSM158 |
|  | | | | | | |
| *Vectors* | | | |  | |  |
|  | pORFM | | | universal in-frame deletion/in-frame fusion vector with GalK-based counterselection  and MCS; *npt*, *galK*, *tetR*, *mobRK2* | | [(6)](#_CTVL001e43ef733da3b4f3ebc4196a21636a63e) |
|  | pORFM-Δ*popZ_Mgr_* | | | vector for chromosomal deletion of *popZ_Mgr_* | | This study |
|  | pORFM-*popZ_Mgr_-gfp* | | | vector for chromosomal insertion of a codon-optimized *gfp* gene (*magegfp*) at the chromosomal *popZ_Mgr_* locus | | This study |
|  | pORFM-*mcherry*-*popZ_Cc_* | | | vector for chromosomal replacement of *popZ_Mgr_* with *mcherry*-*popZ_Cc_* | | This study |
|  | pJH40 | | | transposon vector for random single-copy chromosomal insertion of an anhydrotetracyline inducible expression cassette; pBAM1 with P*_tet_*, *mamC-maggbp-gbp*, P*_Neo_*-TetR, Km^R^, Amp^R^ | | [(7](#_CTVL001ec18adb5cb8143818633f24319064057)[, 9)](#_CTVL0018cbff3591f9b4934b41427d8ecb6f0ed) |
|  | pBAM-P*_tet_*-*popZ_Mgr_* | | | *popZ_Mgr_* cloned into pJH40 via NdeI and BamHI restrictions sites | | This study |
|  | pBAM-P*_tet_-popZ_Mgr_*-*gfp* | | | *popZ_Mgr_-gfp* cloned into pJH40 via NdeI and BamHI restrictions sites | | This study |
|  | pMT009 | | | pBBR1MCS-2 based vector harboring P*_mamAB_*-*mCherry-mamK*, Km^R^ | | [(15)](#_CTVL001bc4c707275db4f0ea61901c8d298d69a) |
|  | pNTPS138 | | | Suicide vector; *oriT*, *sacB*, Km^R^ | | M.R.K. Alley, Imperial College London, unpublished |
|  | pNTPS138-*popZ_Mgr_*-*gfp* | | | Vector for chromosomal replacement of *popZ_Cc_* with *popZ_Mgr_*-*gfp* | | This study |
|  | | | | | | |
| *Primers* | | |  | | |  |
| Lab internal primer number | | | Primer name | | Sequence 5’-3’ | |
| 5 | | | HLINK-oeGFP_fwd | | gctagcctggccgaagccgcgg | |
| 6 | | | HLINK-oeGFP_rev | | tcacttatacagctcgtccatgcccagg | |
| 37 | | | PopZ-oeGFP_up_fwd | | atgagcgacgacaaggcccaacaagaacc | |
| 38 | | | PopZ-oeGFP_up_rev | | cttcggccaggctagcaaggttctctgcgcgatttaccatcttctcg | |
| 39 | | | PopZ-oeGFP_dw_fwd | | cgagctgtataagtgatctctgccggtccgaggacaaggc | |
| 40 | | | PopZ-oeGFP_dw_rev | | atatggcccttcacttcccgctgctcc | |
| 41 | | | PopZ_locus_upper | | cggtgtcgaggaagaggccaaggtcgg | |
| 42 | | | PopZ_locus_lower | | caccttcttacgggcatcgtagcggtcc | |
| 43 | | | PopZ-del_up_fwd | | cctcgcagcagggtaacgaaaccg | |
| 44 | | | PopZ-del_up_rev | | ctaaaggttgtcgctcatggttcgactgtccgatgatcc | |
| 45 | | | PopZ-del_dw_fwd | | atgagcgacaacctttagtctctgccggtccgaggac | |
| 46 | | | PopZ_locus_upper2 | | gtcctatctggatgtggtccgcgacg | |
| 83 | | | oeGFP_BamHI_rev | | cgggatcctcacttatacagctcgtccatgcccagg | |
| 102 | | | popZ_NdeI_fwd | | gggaattccatatgagcgacgacaaggcccaacaagaacc | |
| 103 | | | popZ_BamHI_rev | | cgggatccctaaaggttctctgcgcgatttaccatcttctcg | |
| 408 | | | popZ-Cc_up_fwd | | cgaggccgcccagacgaacaatcc | |
| 409 | | | popZ-Cc_up_rev | | ccttgtcgtcgctcatgtgcggggccgtcgtaaagaggtacg | |
| 410 | | | popZ-Cc_dw_fwd | | cgagctgtataagtgaaacttccgaaccgtcggaaaatcgagg | |
| 411 | | | popZ-Cc_dw_rev | | tcaaggtcggagatggcggtctgg | |
| 412 | | | popZ-Cc_locus_upper | | tcaaacgcccggcgaactggctccc | |
| 413 | | | popZ-Cc_locus_lower | | tcggtggccttgccgtcctcgtcg | |
| 414 | | | mcherry_fwd | | atggtgagcaagggcgaggaggataacatgg | |
| 415 | | | popZ-Cc_rev | | ttaggcgccgcgtccccgagagatacg | |
| 416 | | | popZ-Mgr_up_rev | | cgcccttgctcaccatggttcgactgtccgatgatcccctgg | |
| 417 | | | popZ-Mgr_dw_fwd | | gggacgcggcgcctaatctctgccggtccgaggacaaggc | |

REFERENCES

1. Poindexter JS. 1964. Biological properties and classification of the Caulobacter group. Bacteriol Rev 28:231–295.

2. Poindexter JS. 1978. Selection for nonbuoyant morphological mutants of Caulobacter crescentus. J Bacteriol 135:1141–1145.

3. Falk G, Johansson BC. 1983. Complementation of a nitrogenase Fe-protein mutant of Rhodospirillum rubrum with the nif -plasmid pRD1 containing nif genes of Klebsiella pneumoniae. FEMS Microbiology Letters 19:145–149. doi:10.1111/j.1574-6968.1983.tb00530.x.

4. Heyen U, Schüler D. 2003. Growth and magnetosome formation by microaerophilic Magnetospirillum strains in an oxygen-controlled fermentor. Appl Microbiol Biotechnol 61:536–544. doi:10.1007/s00253-002-1219-x.

5. Schüler D, Uhl R, Bäuerlein E. 1995. A simple light scattering method to assay magnetism in Magnetospirillum gryphiswaldense. FEMS Microbiology Letters 132:139–145. doi:10.1111/j.1574-6968.1995.tb07823.x.

6. Raschdorf O, Plitzko JM, Schüler D, Müller FD. 2014. A tailored galK counterselection system for efficient markerless gene deletion and chromosomal tagging in Magnetospirillum gryphiswaldense. Appl Environ Microbiol 80:4323–4330. doi:10.1128/AEM.00588-14.

7. Borg S, Hofmann J, Pollithy A, Lang C, Schüler D. 2014. New vectors for chromosomal integration enable high-level constitutive or inducible magnetosome expression of fusion proteins in Magnetospirillum gryphiswaldense. Appl Environ Microbiol 80:2609–2616. doi:10.1128/AEM.00192-14.

8. Ptacin JL, Gahlmann A, Bowman GR, Perez AM, von Diezmann, Alexander R S, Eckart MR, Moerner WE, Shapiro L. 2014. Bacterial scaffold directs pole-specific centromere segregation. Proc Natl Acad Sci U S A 111:55. doi:10.1073/pnas.1405188111.

9. Borg S, Popp F, Hofmann J, Leonhardt H, Rothbauer U, Schüler D. 2015. An intracellular nanotrap redirects proteins and organelles in live bacteria. MBio 6. doi:10.1128/mBio.02117-14.

10. Martínez-García E, Calles B, Arévalo-Rodríguez M, Lorenzo V de. 2011. pBAM1: an all-synthetic genetic tool for analysis and construction of complex bacterial phenotypes. BMC Microbiol 11:38. doi:10.1186/1471-2180-11-38.

11. Popp F, Armitage JP, Schüler D. 2014. Polarity of bacterial magnetotaxis is controlled by aerotaxis through a common sensory pathway. Nat Commun 5:5398. doi:10.1038/ncomms6398.

12. Sage D, Donati L, Soulez F, Fortun D, Schmit G, Seitz A, Guiet R, Vonesch C, Unser M. 2017. DeconvolutionLab2. An open-source software for deconvolution microscopy. Methods 115:28–41. doi:10.1016/j.ymeth.2016.12.015.

13. Richardson WH. 1972. Bayesian-Based Iterative Method of Image Restoration*. J. Opt. Soc. Am. 62:55. doi:10.1364/JOSA.62.000055.

14. Lucy LB. 1974. An iterative technique for the rectification of observed distributions. The Astronomical Journal 79:745. doi:10.1086/111605.

15. Toro-Nahuelpan M, Müller FD, Klumpp S, Plitzko JM, Bramkamp M, Schüler D. 2016. Segregation of prokaryotic magnetosomes organelles is driven by treadmilling of a dynamic actin-like MamK filament. BMC Biol 14:88. doi:10.1186/s12915-016-0290-1.

16. Mastronarde DN. 2005. Automated electron microscope tomography using robust prediction of specimen movements. J Struct Biol 152:36–51. doi:10.1016/j.jsb.2005.07.007.

17. Kremer JR, Mastronarde DN, McIntosh JR. 1996. Computer visualization of three-dimensional image data using IMOD. J Struct Biol 116:71–76. doi:10.1006/jsbi.1996.0013.

18. Martinez-Sanchez A, Garcia I, Asano S, Lucic V, Fernandez J-J. 2014. Robust membrane detection based on tensor voting for electron tomography. J Struct Biol 186:49–61. doi:10.1016/j.jsb.2014.02.015.

19. Hanahan D. 1983. Studies on transformation of Escherichia coli with plasmids. J Mol Biol 166:557–580.

20. Schultheiss D, Kube M, Schüler D. 2004. Inactivation of the flagellin gene flaA in Magnetospirillum gryphiswaldense results in nonmagnetotactic mutants lacking flagellar filaments. Appl Environ Microbiol 70:3624–3631. doi:10.1128/AEM.70.6.3624-3631.2004.

21. Evinger M, Agabian N. 1977. Envelope-associated nucleoid from Caulobacter crescentus stalked and swarmer cells. J Bacteriol 132:294–301.

22. Bowman GR, Comolli LR, Zhu J, Eckart M, Koenig M, Downing KH, Moerner WE, Earnest T, Shapiro L. 2008. A polymeric protein anchors the chromosomal origin/ParB complex at a bacterial cell pole. Cell 134:945–955. doi:10.1016/j.cell.2008.07.015.
